# Characterization of the ocular inflammatory response to AAV reveals divergence by sex and age

**DOI:** 10.1016/j.ymthe.2025.01.028

**Published:** 2025-01-17

**Authors:** Alison J. Clare, Philip M. Langer, Amy Ward, Ying Kai Chan, Andrew D. Dick, David A. Copland

**Affiliations:** 1 Academic Unit of Ophthalmology, Translational Health Sciences, University of Bristol, BS8 1TD Bristol, UK; 2 Wyss Institute for Biologically Inspired Engineering, Harvard University, Boston, MA 02115, USA; 3 School of Cellular and Molecular Medicine, University of Bristol, BS8 1TD Bristol, UK; 4 NIHR Biomedical Research Centre of Ophthalmology, Moorfields Eye Hospital, EC1V 2PD London, UK; 5 University College London Institute of Ophthalmology, EC1V 9EL London, UK

**Keywords:** adeno-associated virus, AAV, microglia, T cell, inflammation, age, sex-related differences, eye, gene therapy, retinal degeneration

## Abstract

Progress for ocular adeno-associated virus (AAV) gene therapy has been hindered by AAV-induced inflammation, limiting dose escalation and long-term efficacy. Broadly, the extent of inflammatory responses alters with age and sex, yet these factors are poorly represented in pre-clinical development of ocular AAV gene therapies. Here, we combined clinical imaging, flow cytometry, and bulk sequencing of sorted microglia to interrogate the longitudinal inflammatory response following intravitreal delivery of AAV2 in young (3-month-old), middle aged (9-month-old), and old (18-month-old) *Cx3cr1-creER:R26tdTomato*^*+/−*^ mice of both sexes. Young males and females exhibited a similar dynamic response, with peak inflammation evident at days 10–12 and signs of clinical resolution by day 28. However, the magnitude of the transcriptional response by microglia and adaptive T cell infiltrate differed between sexes. With age, increased and persistent inflammation were observed in both sexes, although old males maintained their microglia transcriptional AAV response signature. Contrarily, females demonstrated greater divergence in their inflammatory response across age, with enriched cellular stress and inflammatory gene expression in older mice and corresponding signs of retinal degeneration. These findings inform crucial sex and age differences for the therapeutic application of ocular gene therapy, highlighting the need to further understand these factors to overcome AAV immunogenicity.

## Graphical abstract

**Figure:**
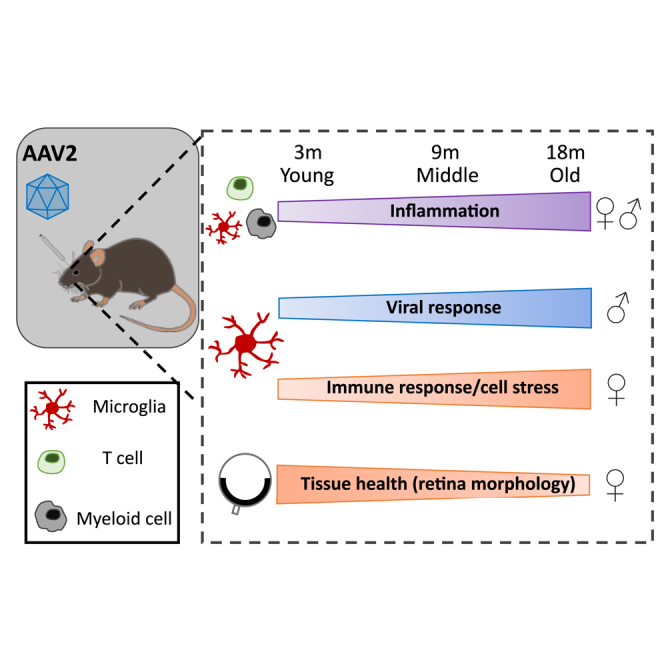

## Teaser

The potential for AAV gene therapy to treat ocular diseases remains limited due to AAV-mediated inflammation. Clare and colleagues characterized in the mouse how age and sex impact the microglia response (resident immune cells in the retina). Of importance, old females display long-term pro-inflammatory changes to microglia and corresponding retinal degeneration.

## Introduction

Recombinant adeno-associated virus (rAAV) has become a leading platform for gene therapy, in part due to the broad tropism, stable non-integrating transduction, and low immunogenicity compared to alternative viral vectors.^1^^,^^2^ Nevertheless, it is evident within pre-clinical and clinical studies that AAV-induced inflammation is a limiting obstacle impacting safety and durable efficacy.^3^^,^^4^^,^^5^^,^^6^ Clinical trials for ocular indications are consistently reporting a dose-dependent inflammation, or gene therapy-associated uveitis (GTAU), which limits dose escalation and therapeutic efficacy.^6^^,^^7^^,^^8^ In severe cases, GTAU has, albeit uncommonly, led to the permanent loss of visual acuity, and long-term efficacy of treatment has only been achieved in 43.6% of ocular gene therapy trials.^9^ Improved understanding of the cellular response to AAV in the eye remains critical to establish effective vector designs and adjunct treatment regimens to control both clinical and subclinical inflammation to improve therapeutic efficacy.

Characterization of the early ocular immune responses to AAV in young animals demonstrates activation of resident microglia,^10^^,^^11^^,^^12^ antigen presentation,^10^ and lymphocyte infiltration.^3^^,^^4^^,^^5^^,^^10^^,^^12^ In preclinical mouse models, the early inflammatory response following intravitreal (IVT) delivery of AAV appears to clinically resolve; however, *ex vivo* assessment highlights subclinical inflammation, with elevated numbers of intraocular immune cells (CD45^+^).^3^ Recognizing that immune responses are divergent according to age and sex,^13^^,^^14^ tolerance for maintaining homeostasis after AAV-mediated alterations to the ocular immune threshold will also differ according to patient background immune status. Sex hormones, incomplete activation of immune-associated genes on the X chromosome, and the Xist ribonucleoprotein complex are contributors to sex differences in immune responses,^13^^,^^14^^,^^15^ leading to a greater susceptibility to autoimmune disease or a more robust pro-inflammatory and adaptive response to viral infection in adult females.^13^^,^^16^ Adult females have a greater adaptive response (T cell activation) across age, while the pro-inflammatory response is greater in adult females, but later in life becomes greater in males.^13^ Despite increased understanding that sex and age can influence immune responses, exploration of the effect that these biological factors exert during AAV inflammation remains unknown. The influence of systemic immunity and disease context on ocular immune response to AAV is highlighted further in recent clinical trials. A higher rate of significant adverse effects, linked to inflammatory events, were reported in patients with diabetic macular edema (INFINITY trial) compared to neovascular age-related macular degeneration (OPTIC trial), despite patients receiving the same therapeutic vector and dose.^17^^,^^18^

To date, studies to understand and develop approaches to control inflammation (e.g., modifying vector—capsid and sequence) have not sought to delineate sex differences.^5^ Where sex is considered, differences in the efficacy of a modified capsid to inhibit myeloid differentiation primary response 88 (MyD88) were identified.^19^ Investigations of the effect of age on AAV immune responses have been neglected.^20^ Understanding how these basic biological factors, age and sex, contribute to shaping the AAV immune response is essential for effective inflammation prevention in all patient populations.

In this study, we use young (3-month-old), middle-aged (9-month-old) and old (18-month-old), male and female *Cx3cr1-creER:R26tdTomato* mice^21^ to describe the longitudinal resident microglia and infiltrating cellular response following IVT delivery of a null AAV2 vector. Confirming previous work,^3^ we observe a dynamic response with a peak in inflammation (days 10–12 post-AAV delivery), followed by spontaneous clinical resolution (late inflammation; days 27–29 post-AAV delivery). In young mice, microglia undergo a significant transcriptional shift at peak inflammation, with greater changes observed in males when compared to age-matched females. Increased age results in an enhanced and extended inflammatory phenotype after AAV2 exposure, and females exhibit greater divergence in their microglia transcriptomic response to AAV across their lifespan. Of note, aged females have an enriched inflammatory microglia gene signature during late inflammation, with corresponding tissue degeneration.

## Results

### *In vivo* and *ex vivo* analyses reveal a dynamic microglial response to IVT injection of AAV2

**Figure 1:**
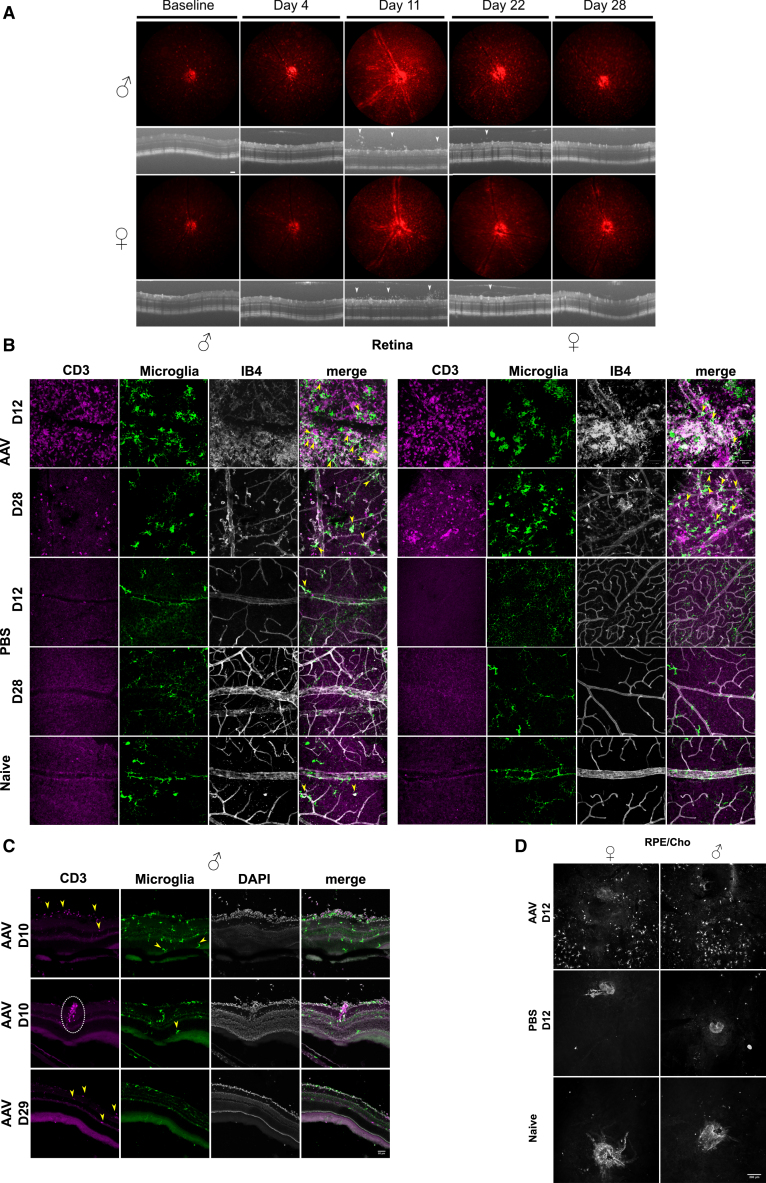
Microglia have a robust and dynamic response to intravitreally injected AAV2 in young animals (3-months-old) (A) Clinical imaging shows representative changes from a male and female eye of microglia (tdTomato fluorescence) distribution and brightness after AAV2 intravitreal (IVT) injections and infiltrating cells to the vitreous (OCT images; white arrowheads). Naive is the pre-injection image. (B) Images of flat mount immunohistochemistry staining for CD3^+^ (magenta) cells, tdTomato^+^ microglia (green), and isolectin B4 (IB4) binding (white). Co-labeling of microglia and IB4 is indicated by yellow arrowheads. Scale bar, 50 μm. (C) Tissue sections stained for CD3^+^ (magenta) cells, tdTomato^+^ microglia (green), and DAPI (white). CD3^+^ cell localization is indicated by yellow arrowheads. White dashed circle highlights cluster of CD3^+^ cells at peak inflammation (day 10). Yellow arrowheads identify microglia migrating to outer retinal layers and sub-retinal space (SRS) on day 10. Scale bar, 50 μm. (D) Immunohistochemistry staining of tdTomato^+^ microglia (white) on RPE/choroid flat mounts.

**Figure 2:**
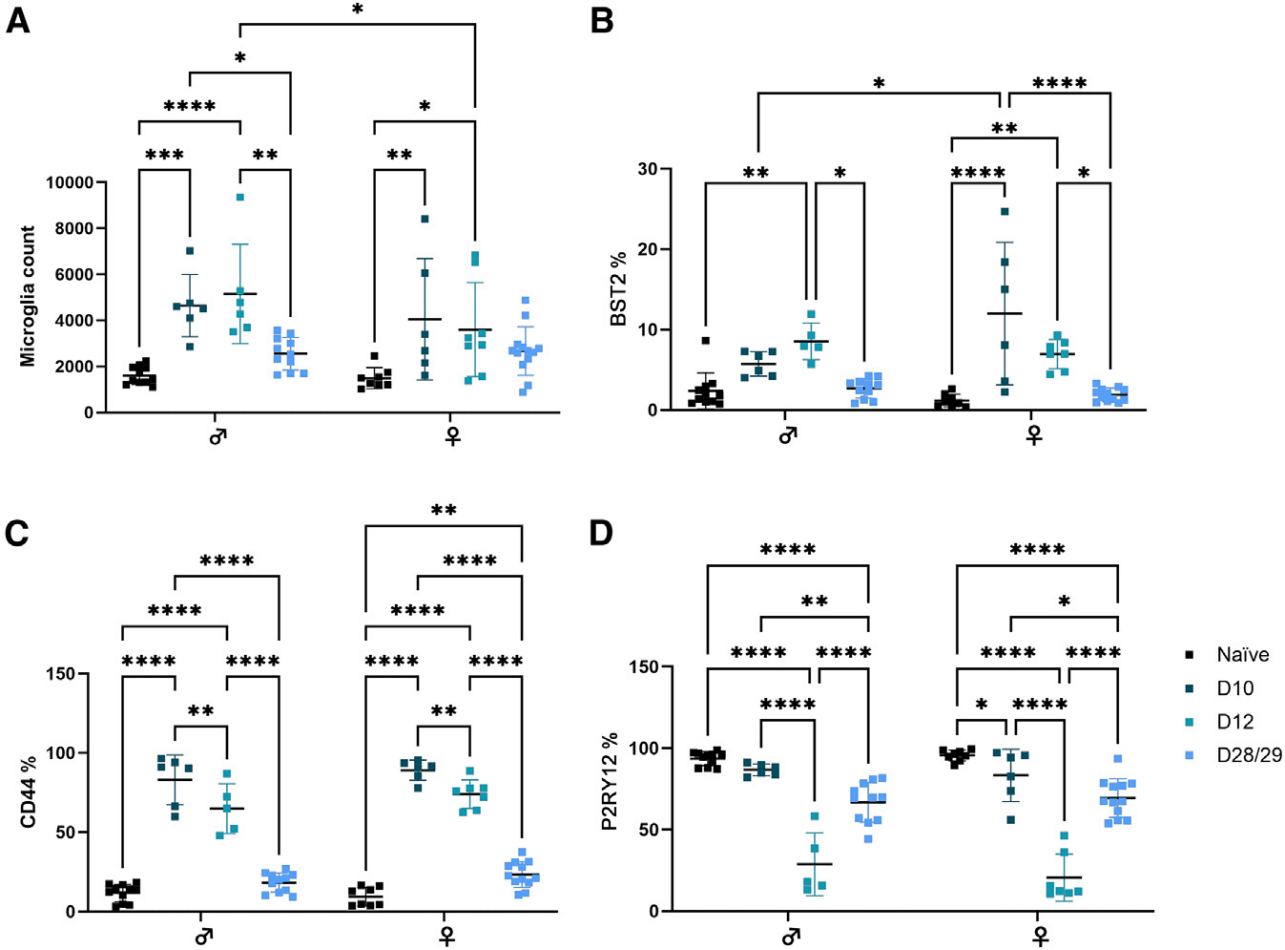
Microglia are increased in number and activated after AAV2 IVT injection in young animals (3-months-old) (A) Graph shows flow cytometric quantification of tdTomato^+^ microglia cells in naive retina compared to days 10, 12, and 28/29 post-AAV injection. Microglia were further characterized by their expression of activation markers percentage, for BST2 (B), CD44 (C), and homeostatic marker P2RY12 (D). All analyzed by two-way ANOVA with Holm-Šídák multiple correction; ∗*p* < 0.05; ∗∗*p* < 0.01; ∗∗∗*p* < 0.001; ∗∗∗∗*p* < 0.0001; graphs are mean ± SD; *n* = 6–13.

In the retina, resident yolk sac-derived myeloid cells (microglia) act as a principal form for immune defense and immune regulation. In the CNS, increasing evidence demonstrates sex-related differences in the microglial compartment, particularly in response to stress (age or disease).^22^ We therefore wanted to assess how microglia respond to IVT-delivered AAV, with consideration of sex. Deploying the *Cx3cr1CreER:R26-tdTomato* (C57BL/6) model,^21^ we evaluated changes to the microglia *in vivo* and *ex vivo* in male and female mice injected IVT with 1E10 genome copies (gc)/eye of AAV2 null (no transgene) virus. *In vivo* fundus and optical coherence tomography (OCT) imaging identified significant alteration to the normal uniform distribution of microglia, which is demonstrated here by increased tdTom brightness and accumulation of microglia at the retinal vasculature (Figure 1A). A consistent peak in inflammation post-IVT AAV occurs at an early time point (days 10–12; Figure 1A), exemplified by perivascular sheathing (PVS) of microglia in 75% of females (18/24) and 80% of males (16/20; no significant difference between sex, *p* = 0.734, Fisher’s exact test) and vitreous infiltrate (as observed by OCT) for 95% of females (22/24) and 85% of males (17/20; no significance between sex, *p* = 0.646, Fisher’s exact test). These changes were significant when compared to PBS-injected eyes (*p* < 0.0001, Fisher’s exact test), in which for both sexes, no infiltrate was observed—males had no PVS (*n* = 6) and 1 (*n* = 7) female eye had mild PVS (Figure S6). Clinical inflammation resolved by a late time point (days 27–29; late inflammation), with microglia returning to a uniform fundal distribution and no inflammatory cells observed clinically in the vitreous (Figure 1A). *Ex vivo* immunohistochemistry analysis of retinal flat mounts confirmed significant inflammation at day 12, with CD3^+^ cells identified in and around vessels and decreasing numbers observed at day 28. Increased microglial activation was apparent by greater binding of isolectin B4, as well as microglia displaying ameboid morphology^23^ (Figure 1B). Migration of microglia to outer retinal layers and the sub-retinal space (SRS) was observed at day 12 post-AAV IVT (Figures 1C and 1D). Single-eye flow cytometric assessment (Figure S4 for gating strategy) revealed an expansion in microglia (CD45^+^CD11b^+^tdTom^+^) number, from 1,602 (392; males) and 1,456 (454; females) to 4,641 (1,353; males, *p* = 0.0003) and 4,048 (2,640; females, *p* = 0.0070) on day 10, when the greatest increase was observed. At late inflammation (days 28/29), microglia numbers are reduced, although only reaching significance in males when compared to peak inflammation (*p* = 0.0038 vs. day 10 and *p* = 0.0297 vs. day 12). Both sexes retain elevated numbers compared to naive, although not significantly by multiple comparison, with 2,527 (819) in males and 2,898 (1226) in females (Figure 2A). Furthermore, phenotypic analysis using a panel of established markers of activation in an endotoxin-induced uveitis model (BST2 and CD44) and of homeostasis (P2RY12)^21^ demonstrate specific changes in the response of microglia to AAV. The percentage of cells expressing BST2 reached a peak increase in females at day 10 (*p* < 0.0001), which was significantly greater than that of males (*p* = 0.0121; Figure 2B), while males had the highest expression and reached significance on day 12 only (*p* = 0.0075). CD44 expression (Figure 2C) was significantly increased at days 10 and 12 in both males (*p* < 0.0001) and females (*p* < 0.0001). Expression of both activation markers was reduced at the late time point (days 28/29), commensurate with a resolving clinical phenotype, although females only retained significant elevation of CD44 expression compared to naive eyes (*p* = 0.0022). Microglia expression of homeostatic marker P2RY12 decreased rapidly during peak inflammation (days 10–12), reaching significance compared to naive eyes at day 12 in males (*p* < 0.0001) and females (*p* < 0.0001). As peak clinical inflammation resolves, expression levels are partially recovered but remain significantly altered at days 28/29 compared to naive eyes in males (*p* < 0.0001) and females (*p* < 0.0001) (Figure 2D). These changes were specific to AAV, as changes to microglial markers were observed only in a dose-dependent manner and not seen in PBS sham-injected eyes (Figure S7).

### AAV-induced transcriptomic changes in microglia are enhanced in male mice

**Figure 3:**
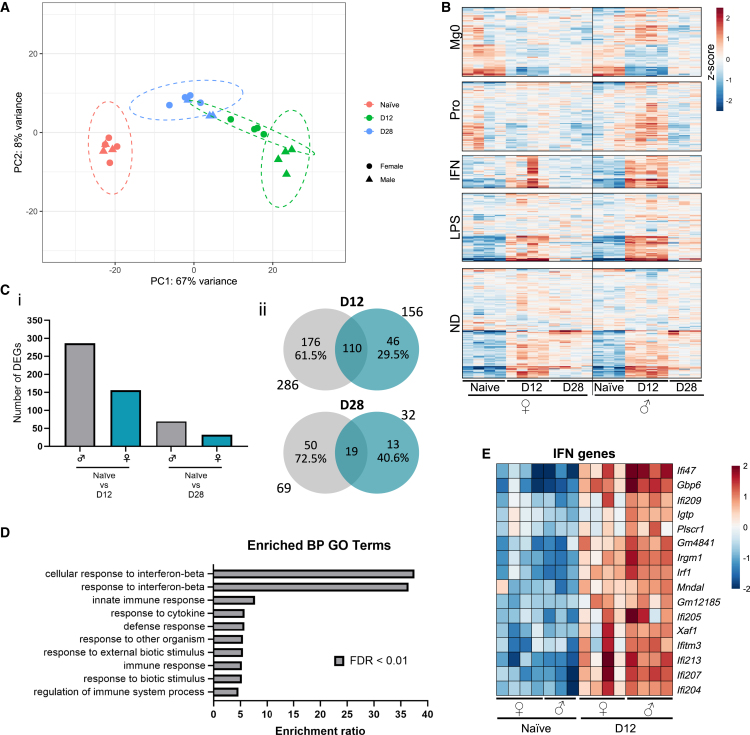
The microglia transcriptomic response to AAV is enhanced in 3-month-old males compared to females RNA sequencing analysis of sorted microglia, naive, day 12, and day 28 post-AAV injection. (A) PCA clustering analysis shows sample clustering, and sample distribution analysis (dashed lines) identifies groupings. (B) Heatmap explores changes in the expression of disease microglia gene modules^24^ for our AAV-stimulated microglia. IFN, interferon response; LPS, lipopolysaccharide response; Mg0, resting state microglia; ND, neurodegeneration-related; Pro, proliferation. (C) Bar graph shows number of differentially expressed genes identified, and the Venn diagrams show genes that overlap for both sexes at day 12 (i) and day 28 (ii). (D) Enriched Gene Ontology (GO) terms (biological processes [BP]) identified among 176 genes differentially expressed in males only at day 12. (E) Heatmap to show *Z* score of differentially expressed genes associated with the IFN GO terms.

Analysis of the microglia response to IVT AAV demonstrate similar and robust kinetics of their response between male and female mice. However, differences in the retained elevation of molecular marker CD44 (compared to their naive counterpart) at late inflammation between males and females, as well as in the difference in response of BST2 expression, indicate a variation in response dependent on sex. We explored the potential for molecular differences in the microglia response to AAV. In the brain, microglia have specific modules of transcriptomic changes depending on the stimuli,^24^ and previous studies have highlighted sex differences in the transcriptomic responses for spinal cord microglia, with perturbed CSF1 responses in females compared to males.^25^ Here, we assessed the complete transcriptomic response of retinal microglia, to probe for sex differences specific to a viral (AAV) response, using bulk RNA sequencing (RNA-seq) of microglia fluorescence-activated cell sorting (FACS) sorted from individual retinas of naive, day 12, and day 28 post-IVT animals. Principal-component analysis (PCA) revealed a significant change in the transcriptome profiles during peak inflammation (day 12; principal component [PC]1 67% variance; Figure 3A) for both males and females. The separation on PC1 analysis was less pronounced for microglia sorted at late inflammation (day 28), indicating recovery toward naive gene expression baseline post-infection. Microglia from female mice showed less separation at peak, with analysis of sample distribution grouping those at peak inflammation (day 12) with samples from the late time point (day 28) post-IVT AAV. Furthermore, examining expression of curated microglia transcriptomic datasets from homeostasis and disease^24^ within our dataset corroborated this finding. Female microglia demonstrated a dampened response at peak across all modules, including those involved in viral-specific responses (proliferation and interferon [IFN] modules; Figure 3B).^24^ Analysis of differentially expressed genes (DEGs) confirmed a greater magnitude of response in males (286 DEGs day 12, Table S1; 69 DEGs day 28; Table S2) at day 12 compared to females (156 DEGS day 12, Table S3; 32 DEGs day 28, Table S4; Figure 3Ci). Of the genes that changed in females, the majority overlapped with male DEGs (70.5% day 12; 59.4% day 28; Figure 3Cii). Gene Ontology (GO) analysis of biological processes (BPs) for the 176 DEGs identified only in males revealed significant enrichment of genes associated with an IFN response (Figure 3D; Table S5), with higher expression at day 12 in males compared to females (Figure 3E). Collectively, these data demonstrate a reduced microglia response to a viral stimulus for females at peak inflammation, despite similarly observed kinetics during the inflammatory response (Figure 2).

### The adaptive inflammatory response to IVT injection of AAV reflects known sex differences in immune cell responses

Following AAV administration and in addition to the observed changes in the distribution of microglia, clinical imaging also demonstrates infiltration of immune cells into the vitreous (Figure 1A). Previous work has shown that AAV-mediated inflammation in the eye predominantly comprises CD3^+^ T cells, indicating an adaptive immune response,^3^^,^^5^ a response mediated by posterior lymphatic drainage after IVT injection.^26^ To understand whether there are sex differences in the recruitment of peripheral immune cells, a single eye flow cytometric gating strategy (Figure S4) was used to further characterize non-microglia CD45^+^ cells, separated based on the expression of B220^+^/CD19^+^ (lymphocyte; B cells), CD11b^+^ (monocytes/granulocytes), and CD3^+^ (lymphocyte; T cells).

**Figure 4:**
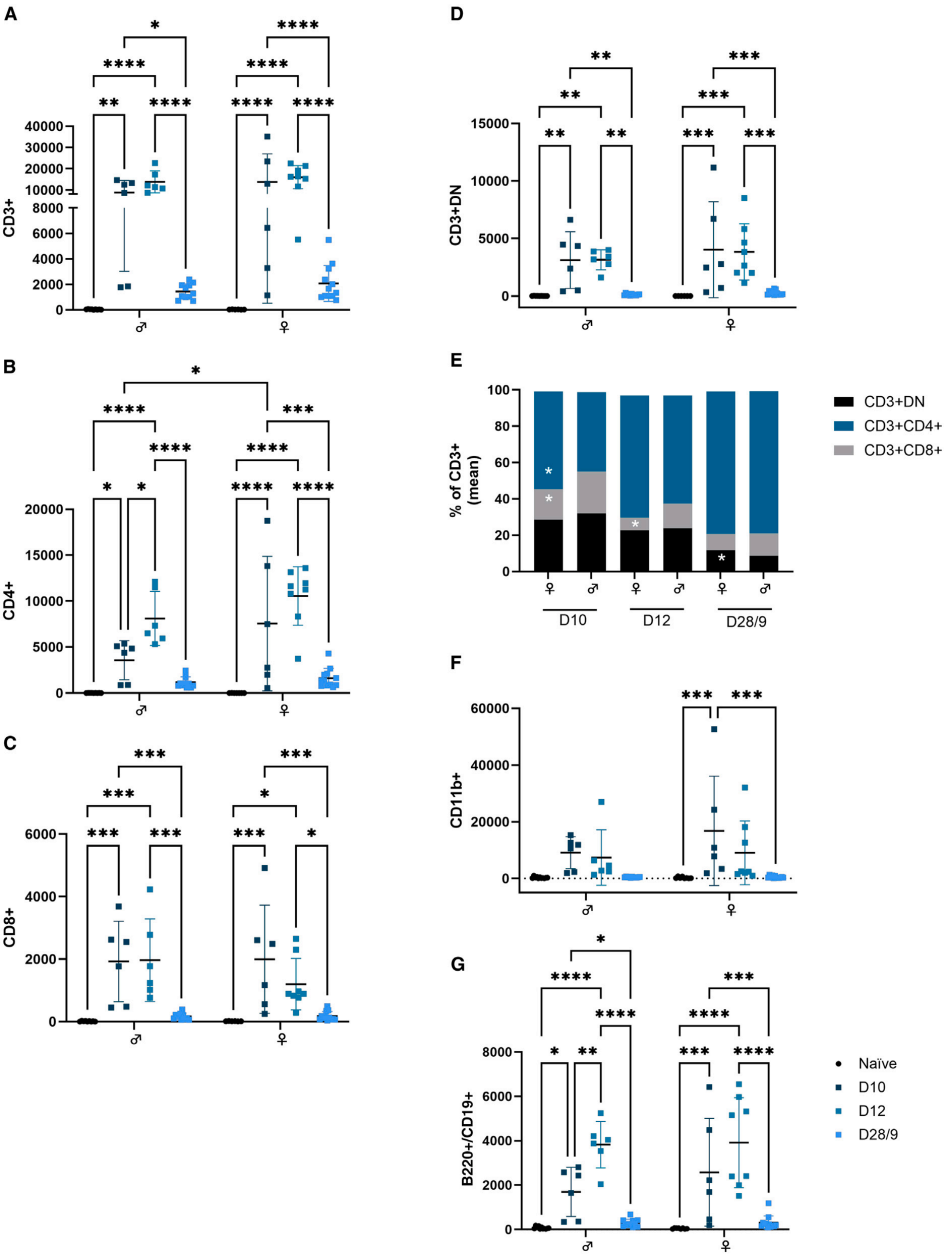
Sex differences in the adaptive response to IVT-delivered AAV in young mice Graphs show non-microglia immune cells quantified from AAV-injected retina by flow cytometry. (A) CD3^+^, (B) CD4, (C) CD8, and (D) CD4^−^CD8^−^. (E) Bar graph shows the proportion (%) of T cell type (CD3^+^) by CD4/CD8 markers. Graphs show (F) non-microglia CD11b^+^ cells and (G) B220^+^/CD19^+^ B cells. *n* = 6–13; all analyzed by two-way ANOVA and multiple correction by Holm-Šídák; ∗*p* < 0.05; ∗∗*p* < 0.01; ∗∗∗*p* < 0.001; ∗∗∗∗*p* < 0.0001.

The number of CD3^+^ were increased at peak inflammation (days 10 and 12), with 13,804 (5,184; *p* < 0.0001) in males and 15,992 (5,459; *p* < 0.0001) in females (day 12), compared to 41 (23) in males and 34 (9) in females (naive; Figure 4A). Further phenotyping of the CD3^+^ population to distinguish CD8^+^ (cytotoxic T cells) and CD4^+^ (T helper [Th]) demonstrates an increase in absolute counts of CD4^+^ (Figure 4B), CD8^+^ (Figure 4C), and double-negative (DN; CD4^−^CD8^−^; Figure 4D) cells at peak inflammation (days 10 and 12) in both sexes, with a significantly higher number of CD4^+^ cells in females (7,564 [7,316]) compared to males (3,573 [2,113]) at day 10 (*p* = 0.0136). Analysis of cell proportions (percentages) revealed significant sex differences for these T cell subsets, with a higher percentage of CD8^+^ cells (23% [4] vs. 17% [6]; *p* = 0.018) in males and CD4^+^ cells (54% 7] vs. 44% [7]; *p* = 0.031) in females at day 10 and higher CD8^+^ percentages in males at day 12 (14 [6] vs. 7 [3]; *p* = 0.016; Figure 4E). At the late time point (days 28/29), CD3^+^ numbers recover significantly compared to peak inflammation (*p* < 0.0001 both sexes) but remain elevated, with females having 2,073 (1,410) and males having 1,446 (595; Figure 4A). As percentages of CD3^+^, both the CD4^+^ and CD8^+^ subsets were increased at the late time point compared to peak inflammation and were similar between sexes. For the DN population, females had a significantly higher proportion of CD3^+^CD4^−^CD8^−^ T cells (12 [3] vs. 9 [3]; *p* = 0.020; Figure 4E); counts were similarly elevated in females, with 254 (195) cells compared to 131 (70) in males, although this did not reach significance.

Non-microglia CD11b^+^ cells were similarly increased during peak inflammation compared to naive (CD11b^+^ 233 [216] males and 112 [36] females), with the highest numbers seen at day 10—9,102 (5,627; not significant by multiple comparison) in males and 16,802 (19,296; *p* = 0.0007) in females (Figure 4F). The greatest number of B220^+^/CD19^+^ (B cells) were seen at day 12—3,826 (1,046; *p* < 0.0001) in males and 3,916 (2,023; *p* < 0.0001) in females (Figure 4G)—significantly increased compared to naive (B220^+^ 95 [63] males, 55 [19] females). By the late time point (day 29), when clinically there was no vitreous inflammation, both CD11b^+^ and B cell numbers were reduced compared to peak, although B cells cell numbers remained elevated compared to naive retinae at 292 (172) in males and 308 (300) in females. No significant sex difference was observed for these cell types.

To understand whether the sex differences in the adaptive CD3^+^ T cell response to AAV were modulated in a dose-dependent manner, we evaluated immune cell numbers following administration of a reduced AAV titer (2E8 gc/eye). At this lower dose, no clinical signs of inflammation are evident in males or females, with only subclinical changes detectable by flow cytometry. Compared to naive, no significant increase in microglia or B cell cell counts at early and late time points are observed, although males exhibit higher CD11b^+^ (non-microglia) cell counts compared to females at day 10. Analysis of CD3^+^ populations reveals elevated numbers in female but not male eyes, shown by increased CD4^+^ (days 10 and 29) and CD8^+^ (day 29) cell counts (Figure S8). These data suggest that sex differences in T cell compartments retain influence during the retinal inflammatory response to AAV and that subclinical alterations to T cell infiltration in the retinal tissue are evident in females, even at low viral titers, despite no clinical inflammation.

### Aged animals have altered and persistent inflammatory response to AAV

Our data demonstrate sex differences in the inflammatory response elicited from AAV in young mice. Recognizing that immune responses are divergent across age and sex,^13^ we next considered how age influences the ocular immune response. Several ocular gene therapies are in development for disorders affecting individuals older than 65 years (e.g., age-related macular degeneration [AMD]).^27^^,^^28^ While clinical trials report AAV immunogenicity and adverse events associated with the therapeutic gene therapies,^8^^,^^29^ to our knowledge, no preclinical assessment on the impact of age has been reported. To determine the effect of age across the lifespan, we evaluated immune responses in 3-, 9-, and 18-month-old male and female *Cx3cr1CreER:R26-tdTomato* mice to mirror the equivalent human ages of young (20 years), middle-aged (40 years), and old (>65 years) adults.^30^

**Figure 5:**
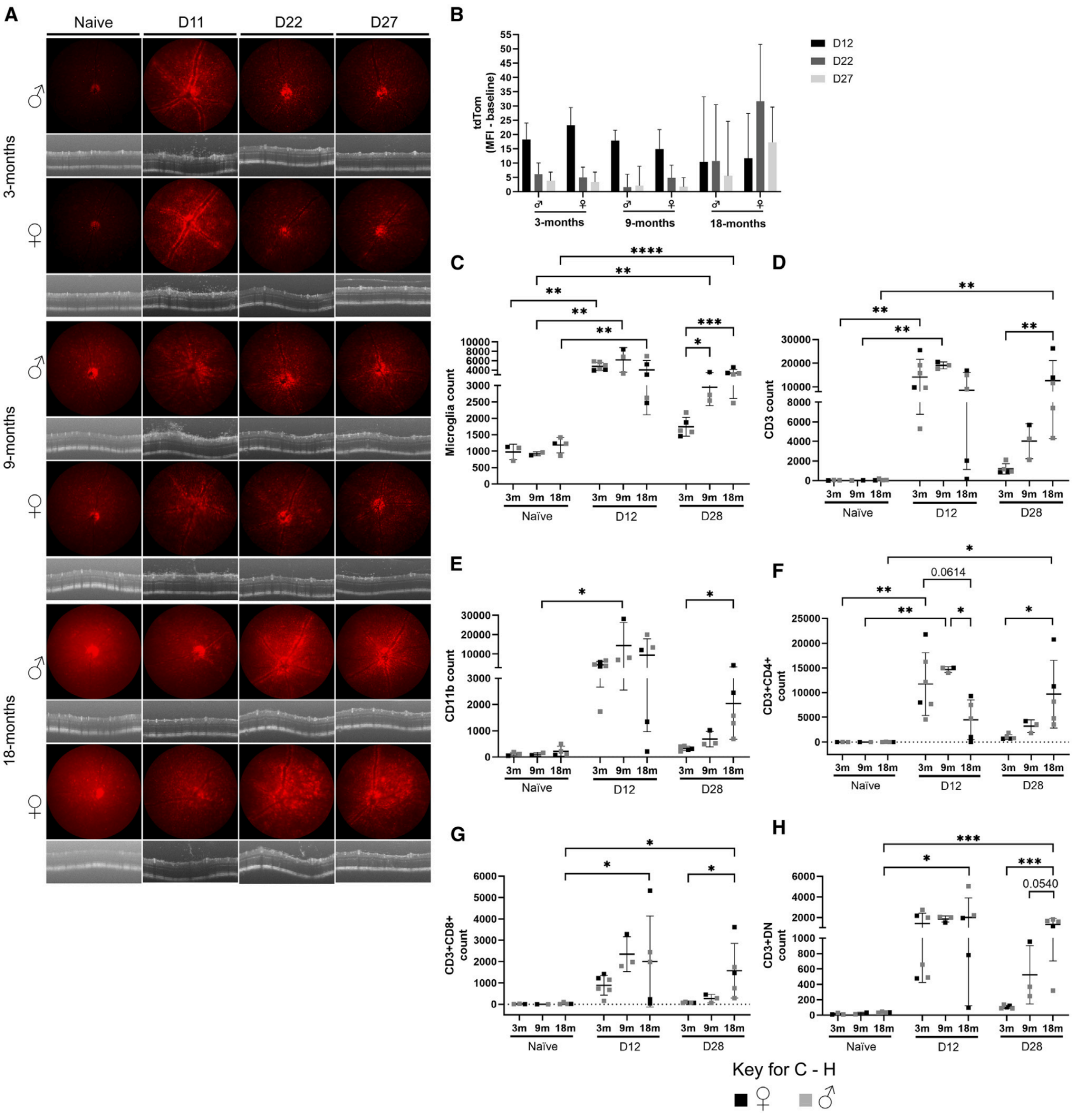
The inflammatory response to IVT-delivered AAV alters with age (A) Representative clinical imaging shows changes to tdTomato expression and distribution in 3-, 9-, and 18-month-old animals (both sexes). Note that baseline autofluorescence increases with age. (B) Bar graph represents mean fluorescence intensity (MFI) of tdTomato at each time point post-AAV IVT injection (days 12, 22, and 27) minus baseline autofluorescence pre-injection (naive). Graphs show quantification of microglia (C), CD3^+^ T cells (D), non-microglia CD11b^+^ myeloid cells (E), CD4^+^ (F), CD8^+^ (G), and CD3^+^CD4^−^CD8^−^ (DN) cells (H). Mixed males and females, *n* = 3–5, on graph, gray dots are males and black dots are females. All analysis carried out by two-way ANOVA with Holm-Šídák multiple comparisons; ∗*p* < 0.05; ∗∗*p* < 0.01; ∗∗∗*p* < 0.001; ∗∗∗∗*p* < 0.0001; graphs are presented as mean ± SD.

We assessed inflammatory kinetics post-IVT AAV (1E10 gc/eye) through analysis of tdTom intensity *in vivo*. Based on earlier analyses in the young animals, we captured changes at the expected peak inflammation (day 12), a mid-time point (day 22, when inflammation begins to subside), and a late time point (day 27, when clinical inflammation is resolved). We observed increased background fundus autofluorescence in naive eyes with age, likely due to age-related changes in the retinal pigment epithelium (RPE), for example, lipofuscin accumulation.^31^ We therefore assessed tdTom mean fluorescence intensity (MFI) subtracted from the baseline (day 0) fluorescence of the eye for comparison across age. The kinetics of clinical inflammation according to tdTom intensity showed a similar trajectory in 9-month-old compared to 3-month-old mice, peaking at day 12 before decreasing at the later time points post-IVT AAV. The 18-month-old mice displayed greater variance in their inflammatory response, with male and female animals exhibiting increased tdTom intensity through to the late time point. This indicates long-term persistence of the inflammatory response and was greatest in the old females, particularly at the mid-time point (day 22) (Figures 5A and 5B).

Flow cytometric analysis of the immune cell populations corroborated the *in vivo* observations. The absolute counts for microglia (Figure 5C) and CD3^+^ T cells (Figure 5D) were increased at an early peak of inflammation across the age groups when compared to age-matched naive animals. At the late time point (day 28), cell numbers recovered toward naive levels in the 3-month-old animals. However, the 9- and 18-month-old mice retained significantly elevated numbers of microglia (compared to 3-month-old mice; Figure 5C) and increased with age. Following the day 12 peak, CD3^+^ T cells persisted at a similar level in 18-month-old mice, remaining significantly higher than both 3-month-old and 9-month-old mice at the late day 28 time point (Figure 5D). Phenotypic analysis of the CD3^+^ T cells revealed lower numbers of CD4^+^ cells in 18-month-old retinas at day 12, compared to both 3-month-old and 9-month-old animals (Figure 5E). No differences were observed for CD8^+^ (Figure 5F) or DN (CD4^−^CD8^−^; Figure 5G) populations at peak inflammation, while all cell types were elevated at the late time point in 18-month-old mice (compared to 3-month-old mice; Figures 5E–5G). The non-microglia CD11b^+^ monocyte population increased in all ages at peak disease, reducing in number by the late time point, but remaining elevated in aged mice (Figure 5H). Collectively, these data demonstrate that an altered and persistent inflammatory response is associated with age.

### The microglia response is dysregulated with age and at a greater extent in females

The contribution of microglia in disease associated with age is dichotomous: protective homeostatic roles described on the one hand, yet dysregulated function and phenotype implicated with greater disease severity or progression on the other hand.^32^^,^^33^^,^^34^ The observed alteration to the immune response and accompanied with a persistent elevation in microglia numbers led us to investigate how the molecular response of the resident microglia differs by sex and age. Accordingly, FACS-sorted tdTomato^+^ microglia isolated from single retinae of naive, day 12, and day 28 post-IVT AAV eyes in 3-, 9-, and 18-month-old male and female mice were processed and analyzed using bulk RNA-seq.

Comparing 3- to 9-month-old animals, we observed greater separation of day 12 post-IVT AAV microglia, compared to naive, for both males and females (except one outlier for 9-month-old males; Figure S9), indicating a greater magnitude of the inflammatory response. Of note, females had a comparable response to males at 9 months of age (Figure S9). Accordingly, direct comparison of female microglia in 3- and 9-month-old mice at day 12 post-AAV IVT revealed 434 DEGs (Table S6), and GO term (BP) analysis identified a significant association with IFN response (*Igtp*, *Ifi47*, *Ifit1*, *Ifi203*, *Gbp3*, *Gbp2*, *Ifi205*, *Iigp1*) and cell division (*Ccnd1*, *Kif2c*, *Top2a*, *Cdca8*, *Cenpe*) among the upregulated genes (Table S7). For males, the same age comparison at day 12 only demonstrates 67 DEGs (Table S8), with upregulated genes significantly and primarily associated with cell division (*Ube2c*, *Cenpa*, *Top2a*, *Cdca8*; Table S9). At the late time point, only a small number of DEGs were identified for males (1 DEG; Table S10) and females (14 DEGs; Table S11). These data demonstrate significant changes in the response of female microglia with age. Comparatively male microglia at 9 months of age display enhanced but similar gene changes compared to their younger counterparts.

**Figure 6:**
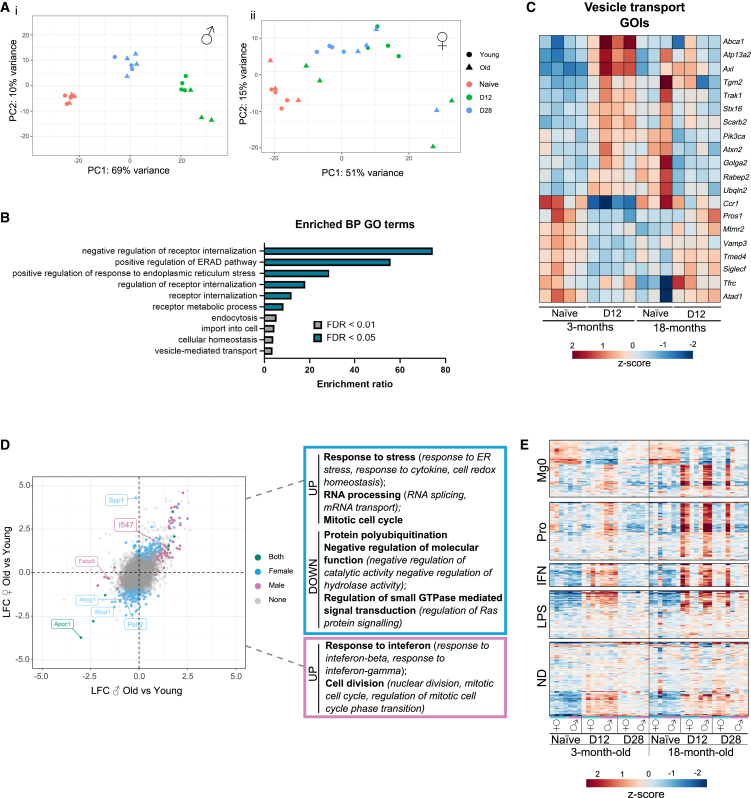
Aged female microglia undergo the greatest alterations in microglia transcriptomic response to IVT injection of AAV (A) PCA clustering analysis identifies similar clustering of samples according to naive and day 12 or day 28 post-AAV injections in males (i), while old females have more variability in the day 12 and day 28 samples (ii). (B) GO (BPs) enrichment analysis of genes differentially expressed by age at day 12 in females. (C) Heatmap to show expression of genes of interest identified within the vesicle-mediated transport enrichment terms. (D) Scatterplot to show log-fold change (LFC) between old (18-month-old) and young (3-month-old) males vs. females. Differentially expressed genes are colored according to change in expression in either old males, old females, or both. Boxes show GO (BPs) enrichment terms identified among the up- or downregulated genes for old females (blue) or males (pink). (E) Heatmap explores changes across age and sex for the expression of disease microglia gene modules^24^ for our AAV-stimulated microglia. LPS, lipopolysaccharide response; Mg0, resting state microglia; ND, neurodegeneration-related; Pro, proliferation.

Recognizing altered kinetics of the inflammatory response (clinical and cellular) observed in the 18-month-old animals (Figure 5), our analysis next sought to examine the differences in gene expression in this age group. PCA comparing 18- to 3-month-old males demonstrates that the microglia transcriptomes clustered closely into groups of naive, day 12, and day 28 post-IVT AAV samples (PC1 69% variance; Figure 6Ai), indicating similarity in the viral response across age. Accordingly, only 12 DEGs at day 12 (Table S12) and 1 DEG at day 28 (Table S13) were changed with age. Comparatively, the clusters were less defined for female microglia, where we observed greater variation in transcriptome changes at day 12 for 18-month-old animals, while aged day 28 samples clustered more closely with 3-month-old day 12 (PC1 47% variance; Figure 6Aii). Analysis of genes that change differentially from naive eyes and by age identified 108 DEGs at day 12 (Table S14) and 14 DEGs identified at day 28 (Table S15). GO term (BP) analysis of the 108 DEGs revealed significant enrichment for genes involved in endocytosis and vesicle-mediated transport (Figures 6B and 6C; Table S16).

To interrogate the age differential microglia signatures in response to AAV, we next compared microglia at day 12 post-IVT AAV isolated from the 3- to 18-month-olds in male and female animals. Our analysis revealed that microglia from females had 278 DEGs (Table S17) and males only had 69 DEGs (Table S18) when comparing old and young, and there was a greater dysregulation of microglia response to a viral stimulus with age in females (Figure 6D). GO term (BP) analysis revealed an upregulation of IFN response (*Gbp2*, *Gbp5*, *Gbp6*, *Gbp7*, *Ifi47*, *Ifit1*) and cell division (*Ccnd2*, *Mad2l1*, *Ndc80*, *Stmn1*, *Top2a*, *Ube2c*) in old males indicating an expected but enhanced response to the virus (Figure 6D; Table S19). Downregulated genes in old males were associated with fatty acid processing (*Apoc1*, *Fabp5*), although no term reached significance by false discovery rate (FDR) (Table S20). For old females, GO term enrichment analysis revealed a significant association of downregulated genes involved with protein polyubiquitination (Table S21), which is important to protein trafficking and degradation,^35^ while upregulated genes were linked with cellular stress response terms (Figure 6D; Table S22). When we examined the 3- and 18-month-old microglia samples in the context of the curated activation modules,^24^ a prolonged upregulation of the neurodegeneration-related (ND) and lipopolysaccharide (LPS) modules was observed at day 28 in aged samples, with prominence in females (Figure 6E). Concurrently, direct comparison of microglia at day 28 post-AAV IVT identified 213 DEGs between young and old females (Table S23). Of these DEGs, upregulated genes were enriched in immune response terms (Table S24), with overlap in the LPS (*Cd44*, *Cybb*, *Tnfaip2*, *Cd40*, *Vcam1*, *Xdh*, *Slfn5*, *AB124611*, *Cxcl13*, *Slamf8*, *Mapkapk2*, *C3*, *Ifitm3*) and ND (*Ch25h*, *Cst7*, *Itgax*, *Lgals3bp*, *Ptger4*, *Rxrg*, *Tlr2*, *Cd274*, *Prr5l*, *Cpd*, *Axl*, *Ccl6*) modules. Only five DEGs were identified for old males (*Naalad2*, *Cp*, *Casp12*, *Arsg*, *Tmem167*; Table S25). These data demonstrate an altered microglia response to AAV with age, and male microglia demonstrate an enhanced response during “peak” inflammation. However, female microglia display substantial dysregulation highlighted by upregulated GO terms in stress response during early inflammation and an enhanced chronic inflammatory phenotype.

### AAV stimulus causes retinal thinning in aged females

**Figure 7:**
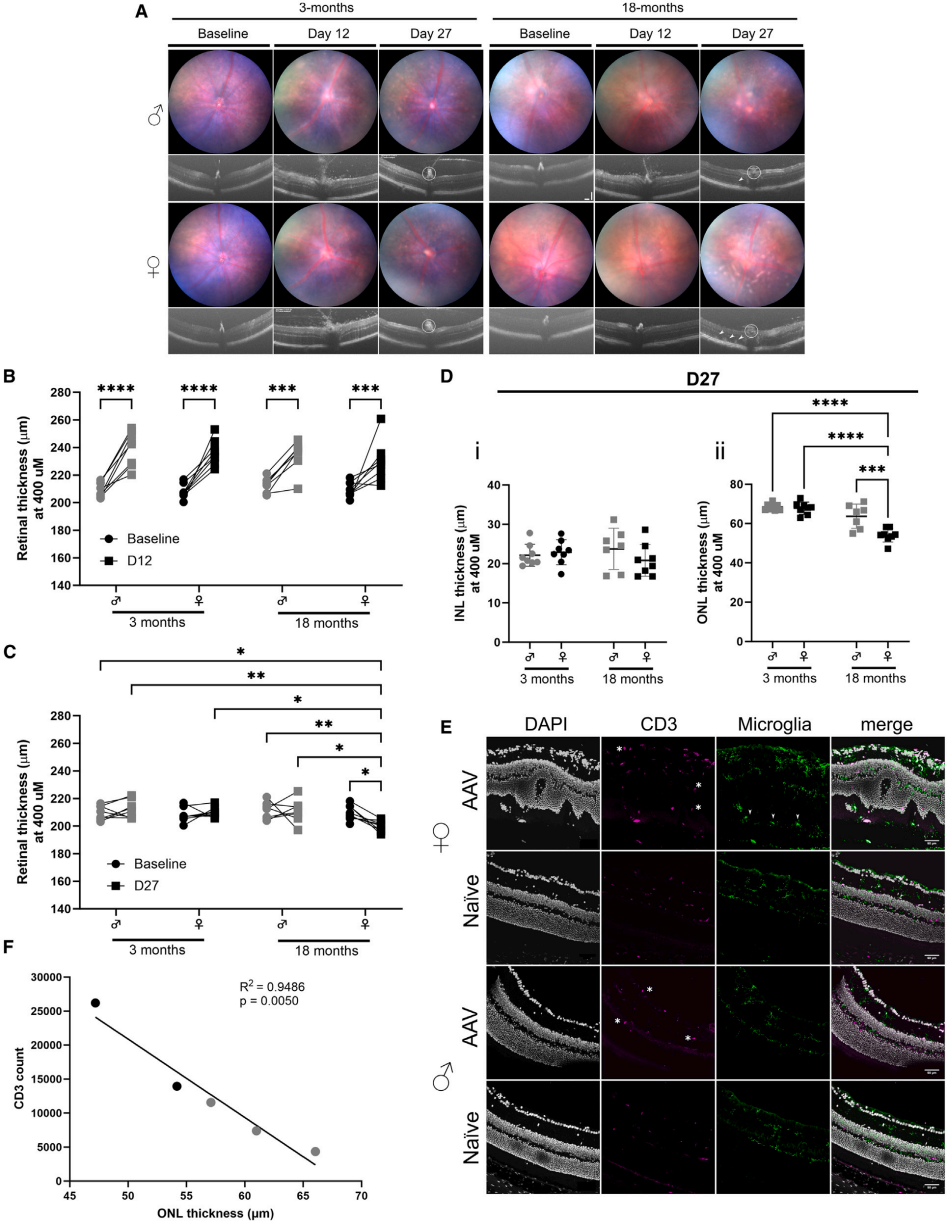
Old female retinas degenerate after IVT delivery of AAV (A) Representative clinical images (fundal and OCT) to show changes after AAV injection. White circle in OCT image indicates thickening of optic nerve head, and white arrows indicate hyper-reflective foci in the outer nuclear layer (ONL). (B and C) Graph shows total retina thickness at baseline (pre-injection) compared to day 12 (B) and baseline compared to day 27 (C) post-AAV injection. (D) Graphs show inner nuclear layer (INL) thickness (i) and ONL thickness (ii) at day 27 post-injection. (E) Immunohistochemistry staining of CD3^+^ (magenta) cells, tdTomato^+^ microglia (green), and DAPI staining of nucleus (white) on sections from 18-month-old male and female mice at day 28 post-AAV injection. Bright CD3^+^ (magenta) cells are indicated by white asterisk. Microglia (green) migration to the SRS is indicated by white arrowheads. (F) Simple linear regression analysis of ONL thickness measured from clinical images on day 27 against total number of CD3^+^ cells quantified from the same eye on day 28 (*n* = animal). Black indicates females and gray indicates males. All analysis carried out by two-way ANOVA with Holm-Šídák multiple comparisons; ∗*p* < 0.05; ∗∗*p* < 0.01; ∗∗∗*p* < 0.001; ∗∗∗∗*p* < 0.0001; graphs are mean ±SD. For (B)–(D), *n* = eye.

Having observed a persistent inflammatory phenotype in the retina of aged animals to IVT AAV delivery and exhibiting a degenerative molecular phenotype in aged female microglia, we next wanted to evaluate the effect on retinal morphology, as a surrogate to tissue health. OCT images provide an *in vivo* cross-sectional high-resolution image through the retina, allowing us to monitor longitudinal changes in posterior segment morphology and pathology.^36^ Here, we assessed OCT images in our 3-month- and 18-month-old animals. At peak inflammation (day 12), hyper-reflective spots were observed in the vitreous, indicating infiltration of immune cells, although they appeared to a lesser extent in 18-month-old animals, concurrent with our observation of a dampened early response with age. Fewer spots are apparent by day 28, although thickening of the optic nerve head is consistent with a continued infiltrate at this site. Hyper-reflective foci in the outer nuclear layer (ONL) appear in aged animals by day 27 and to a greater extent in females (Figure 7A). The clinical presentation of these hyper-reflective foci were observed in all 18-month-old female tdTom mice and to a greater extent compared to males (Figure S10). This was confirmed in C57BL6/J mice aged 11 and 12 months old, with 11/12 (92%) females presenting with a clinical change compared to 6/12 (50%) males (*p* = 0.0686, Fisher’s exact test; Figure S10). No changes were observed in PBS-injected eyes at day 28 (Figure S10). Analysis of the retinal thickness from OCT images allows for a measure of potential tissue inflammation or resultant damage/degeneration. Measurement includes total retinal thickness (TRT), from retinal nerve fiber layer to the RPE layer (inner to outer retina), at baseline, peak inflammation (day 12), and late inflammation (day 27). Compared to the baseline (pre-injection) image, TRT was significantly thicker for all ages and sexes at peak inflammation (Figure 7B). By late inflammation, TRT had recovered close to baseline levels for 3-month-old mice and 18-month-old males. Old females had significant thinning of TRT compared to their baseline and to age-matched males, young males, and young females at the same time point (day 27; Figure 7C). OCT images can be further segmented to specific retinal layers (Figure S11). Significant ocular inflammation results in the loss of photoreceptors, demonstrated by thinning of the ONL.^37^ To establish where retinal cell loss had occurred, we assessed the thickness of the ONL and inner nuclear layer (INL; bipolar cells, amacrine cells, interplexiform neurons, and Müller glia), as a comparator, at late inflammation (day 27). No changes were observed in INL thickness (Figure 6Di), while old females had significant thinning of the ONL compared to age-matched males (*p* = 0.0002), young males (*p* < 0.0001), and young females (*p* < 0.0001; Figure 6Dii), indicating photoreceptor loss*. Ex vivo* analysis of thickness in 3- and 12-month-old C57BL6/J wild-type mice confirmed an increased risk of thinning in old females, although the magnitude of degeneration was not as great at 12 months compared to 18 months (Figure S10).

The clinical presentation of hyper-reflective foci was identified as folds in the retina *ex vivo* and correlated with greater inflammation (presence of CD3^+^ cells) and migration of microglia to the SRS (Figure 6E). In our earlier flow cytometric analyses (Figure 3), we identified a greater retention of CD3^+^ cells at late inflammation (day 28) in old animals, with the mean number of cells equivalent to those seen at peak inflammation for young animals (day 12). We therefore employed these data to investigate correlation of tissue degeneration and to late (day 28) infiltrating T cells. Plotting the number of CD3^+^ immune cells at late inflammation in aged mice against the respective ONL thickness identified a significant inverse correlation (*p* = 0.0050; Figure 6F). These data indicate a degenerative effect of AAV IVT, with increased risk in aged females and correlating to the retention of high numbers of CD3^+^ cells at late inflammation (day 28) and migration of microglia to the SRS.

## Discussion

AAV immune response and subsequent toxicity have arguably limited gene transfer and therapeutic efficacy, making the prevention of immunogenicity a key focus of gene therapy research.^20^^,^^38^^,^^39^ However, the inclusion of age and sex as potential determinants of immune response has been largely neglected in preclinical development, despite the known divergence. Here, we provide evidence and a description of changes to characterize the influence of these factors on the retinal inflammatory response to IVT delivery of AAV2. We identify sex and age differences in the innate (resident microglia) and adaptive response (lymphocyte infiltrate) to AAV2. Sex differences in the microglial response are retained with age, although for both sexes, age leads to an enhanced and chronic response to viral stimulus. Old females display the greatest divergence in the microglia response to AAV and display morphological evidence of retinal degeneration. Our data highlight the importance of continued interrogation of age and sex to improve our understanding and develop future strategies to prevent inflammation and produce safer and effective gene therapies for all target patient groups.

Clinically, the inflammatory response to AAV2 stimulus in young animals has similar presentation between male and female mice. We consistently observe an early peak in ocular inflammation (days 10–12) demonstrated by PVS and vitritis (cellular infiltrate), which does spontaneously subside by day 28 (Figure 1). While inflammation appears to be resolving clinically by day 28, *ex vivo* analysis reveals that P2RY12 expression in microglia is only partially recovered and CD45^+^ infiltrate remains elevated (Figure S8), supporting the notion of a persistent and subclinical change in the ocular immune environment. These findings are consistent with previous observations of GTAU kinetics in response to IVT delivery of AAV2,^3^ indicating a robust and definitive inflammatory effect of AAV2 in the eye, which is specific to AAV2-injected eyes compared to buffer-controlled sham injection (Figure S7). Further longitudinal studies may inform whether inflammation fully resolves and returns to naive levels or whether these alterations to the immune threshold persist.

Here, we chose to analyze the effects of AAV2, which, along with AAV2.7m8 (derived from AAV2), make up the current choice in clinical trials for IVT-delivered gene therapies.^27^ However, whether these findings are specific to AAV2 or widely applicable to other AAV capsid serotypes is called into question. Wiley et al.^40^ provide a detailed comparison of AAV capsids and route of delivery to the eye and the ocular inflammatory response in rats. These data demonstrate subclinical inflammation in AAV2 and AAV6 vectors, while AAV1, AAV8, and AAV9 have mild to no inflammation. Importantly, however, only AAV2 and AAV6 reliably transduced the retina. While Wiley et al.^40^ did not observe a correlation with GFP expression and inflammation, a study of suprachoroidal injected AAV8 in non-human primates indicated that GFP expressed scleral fibroblasts as a driver of adaptive inflammation,^41^ which could suggest that efficacy of infection along with transgene product expression drive a greater inflammatory response. Furthermore, and in agreement with our findings (Figure 1), Wiley et al. demonstrated the migration of Iba1^+^ cells (microglia and circulating monocytes) to the outer retina after IVT injection of both AAV2 and AAV6, suggesting activation and changes to microglia function during infection. However, further studies to directly compare microglial changes in response to different AAV serotypes across age and sex would be required to confirm whether there is a common response.

Despite the similarities in clinical presentation between males and females, our analysis of known inflammatory microglia markers^21^ suggested divergence in molecular changes behind the response (Figure 2). Transcriptomic profiling of microglia revealed similar molecular changes, with 70.5% of female-only DEGs at day 12 overlapping with male DEGs. However, males had an enhanced response with the upregulation of viral-specific gene expression changes in IFN response and proliferation compared to females (Figure 3). The magnitude of change could, in part, be explained by sex differences in the baseline naive expression of viral-responsive genes. For example, IFN-associated genes, including IFN regulatory factor (*Irf1*), are enriched in naive female brain microglia^42^^,^^43^ and subsequently are found to be more responsive to IFN stimulation.^42^^,^^44^ Indeed, in our data, naive female microglia display higher expression of the IFN-related genes compared to males (Figure 3E). However, sex comparisons of these genes at day 12 still demonstrate much higher expression for these genes in male microglia (Figure 3E), and together these transcriptomics data support a more prominent role for microglia in the male AAV inflammatory response.

While transcriptomic analyses identify a dampened microglia response in females, clinical presentation remains similar, suggesting that inflammation is achieved through divergent mechanisms and cell types between males and females. Female mice have an expanded resident T cell population in the periphery,^13^^,^^45^ which expresses higher levels of inflammatory and antiviral genes upon stimulation.^46^ Furthermore, in a mouse spinal nerve injury model, allodynia is preferentially mediated by infiltrating T cells in females, while in males, microglia are the main drivers in this pain response.^45^ In young mice, we observe sex differences in the proportion of different CD3^+^ T cell subsets (CD4^+^, CD8^+^ or CD4^−^CD8^−^) and a more rapid increase in CD4+ cells during peak inflammation days 10–12 (Figure 4). Importantly, even with delivery of a lower, “non-inflammatory” AAV2 dose, only female mice display an increased number of infiltrating T cells to the retina, with elevated CD4^+^ counts at early/peak inflammation and CD4^+^ and CD8^+^ cells both increased at the late stage (Figure S6). These findings could indicate a greater importance for infiltrating T cells in regulating the AAV2 inflammatory response in females. Further analyses of the inflammatory response such as immunophenotyping T cell subsets (e.g., Th1, Th2, Th17), using targeted pharmacological interventions to inhibit the adaptive infiltrate or conversely ablate the microglial compartment, and examining other retinal antigen-presenting cells (e.g., Müller glia) will inform pathways where sexual divergence lies within the mechanisms that ultimately drive a similar clinical response.

The ocular inflammatory response to AAV2 is altered with age, with older males and females demonstrating prolonged and persistent elevation of immune cells (microglia, CD45^+^CD3^+^, CD45^+^CD11b^+^; Figure 5). However, the transcriptomic changes observed in the microglial response to AAV2 across age are more distinct in female mice (Figures 6 and S9). The transciptomic response to AAV2 for aged male microglia was similar compared to young males, although the magnitude of change in gene expression is greater during peak inflammation for 18-month-old mice, suggesting an enhanced inflammatory response (Figure 6). Female mice similarly have an enhanced response at 9 months of age, comparable to age-matched male counterparts (Figure S9). However, by 18-months, their microglia exhibit a distinct and dysregulated inflammatory response and prolonged expression of a pro-inflammatory microglia gene signature (Figure 6). Age-related sex differences in the functional phenotype of microglia is known in the CNS, where aging female microglia have a greater increase in the expression of genes associated with inflammatory processes and an activated state.^22^^,^^47^^,^^48^ In mouse models of Alzheimer disease (AD), the switch to a disease-associated gene signature in microglia occurs earlier in females^49^ and is more pronounced.^50^ These findings support a key role for age- and disease-related microglial changes in driving the sex bias for AD prevalence and pathology.^22^ Similarly, in response to IVT AAV2, we observe a significant degenerative response (thinning of the retina) in aged females only (Figure 7), which also maintains a dysregulated inflammatory microglia signature and accumulation in the SRS at the late inflammation time point. These observations highlight the risk and potential of adverse reactions to AAV gene therapy in women and emphasize a clear rationale and need for further evaluations to delineate the mechanisms responsible for such sex-specific disease-associated differences. One hypothesis for the greater variance across age in females could be the change in sex hormones across the lifespan. Certainly, estrogen signaling is important for anti-inflammatory and neuroprotective signatures in microglia in neurological disease, with both reduced hormone levels and reduced receptor expression implicated.^51^ Mice enter reproductive senescence between 9 and 12 months,^52^ and a reduction in basal levels of estradiol is seen from 10 months.^53^ Although we did not assess hormones in this study, the correlation of age-related changes and the anti-inflammatory role of estrogen signaling in the microglia response would warrant further investigation in the context of the dysregulated microglia response to AAV2 in old females. Assessment via targeted interventions (pharmacological or genetic) to modulate the functional phenotype of resident microglia and/or peripheral immune cells or manipulate sex hormone expression will build our understanding of how the identified transcriptomic changes link and contribute to the degenerative phenotype.

Collectively, our data demonstrate significant sex and age effects on the immune response following IVT delivery of ocular gene therapy. These findings have implications for relative AAV safety depending on sex and age. Women are disproportionately affected by the majority of ocular diseases, including age-related conditions such as AMD and glaucoma.^54^^,^^55^ Gene therapy to treat AMD is an emerging approach, with 13 AAV-delivered treatments in clinical trials^56^ and an ongoing pre-clinical focus on developing gene therapies for glaucoma.^57^ Although the reporting of patient sex in the publication of clinical trial outcomes is improving, data are not sex disaggregated.^8^^,^^29^^,^^58^ Sex differences in transgene expression have been observed in pre-clinical studies with AAV delivery to liver or systemically,^59^^,^^60^ with females showing lower levels of transgene expression, linked to both sex hormones^59^ and a more robust humoral immune response in females.^60^ Our study demonstrates that aging patients, in particular, women receiving ocular gene therapy, could be at a higher risk of developing chronic inflammatory effects and associated adverse effects. These findings highlight the need to stratify data in both pre-clinical studies and clinical trials to best identify therapeutic efficacy and mitigate risk for different patient populations. Moreover, further investigations of the immune pathways and cellular responses with these biological backgrounds (age and sex) will be crucial for the development of prevention strategies to make AAV gene therapy safe and effective for all patients.

## Materials and methods

### Animals

*Cx3cr1CreER:R26-tdTomato* mice on a C57BL/6J background were originally provided by Clemens Lange (University of Freiburg, Freiburg, Germany). Homozygote breeding colonies were established at the University of Bristol, and offspring crossed with C57BL/6J mice to generate heterozygotes were used here for experiments. To activate tdTomato expression locally in the retinal microglia, animals were dropped 3 times daily (every 2–3 h) with 5 mg/mL tamoxifen (T5648; Sigma-Aldrich, Poole, UK) in corn oil (C8267; Sigma-Aldrich) for 3 days, followed by a washout period of at least 4 weeks, before using animals in experiments. This allowed for increased specificity of microglial labeling.^21^ This mouse strain was confirmed negative for the Rd8 mutation.^21^^,^^61^ Except for one experiment on aging animals (see Figure 4), equal numbers of sex-matched mice were used and are identified in the data presentation.

C57BL6/J animals of 3 and 11–12 months old, with equal numbers of males and females, were acquired from Charles River Laboratories (Harlow, UK). Mice were housed at the University of Bristol Animal Services Unit under specific pathogen-free conditions with food and water *ad libitum*. All procedures were approved by the University of Bristol Ethical Review Group. Work was conducted in accordance with the UK Home Office license (PPL 9783504) and the Association for Research in Vision and Ophthalmology Statement for the Use of Animals in Ophthalmic and Visual Research.

### *In vivo* procedures

Prior to any procedure, pupils were dilated using topical tropicamide 1% w/v and phenylephrine 2.5% w/v (Minims; Chauvin Pharmaceuticals, Romford, UK), before anesthesia with 2% isofluorane (Piramal Critical Care, West Drayton, UK). AAV was administered by IVT injection (2 μL/eye), using a 33G needle on a microsyringe under direct visualization (Hamilton Company, Reno, NV). Retina fundal imaging and OCT scans were captured using Micron IV (Phoenix Research Laboratories, Pleasanton, CA). For tdTomato imaging, a 550/25-nm band-pass excitation and 590-nm long-pass emission filter were used (Edmund Optics, Barrington, NJ). Gain was set to +3 dB and the frames per second (FPS) to 15, or +12 dB and 2 FPS for bright-field and tdTomato fluorescence imaging, respectively. The retinal thickness and MFI (tdTomato) were determined using Fiji.^62^ Mice were assigned to groups (naive, day 12, and day 28 post-AAV IVT) in a constrained randomized order within blocks, depending on cage allocations and the litter they were derived from. Experiments in the young animals were made up of groups of males and females derived from the same litters. For the aging experiments, treatments were split across male and female littermates for 9-month-old animals. Of the 18-month-old animals 4/12 males were littermates, and 6/11 females were used. The remaining animals were made up of animals aged from several different litters.

### Plasmid cloning and AAV production

In this study we used an io2 vector with a TLR9 inhibitor oligonucleotide sequence. TLR9, an immune sensor for viral DNA, plays a key role in detecting AAV genomes and triggering innate immune responses.^5^ Our prior work with IVT administration of an AAV io2 vector (AAV.CMV.GFP.WPRE.io2) in mice identified that despite the TLR9 inhibitory oligonucleotide sequence, intraocular inflammation is still observed, suggesting that multiple factors contribute to GTAU. Here, we sought to use a similar system for this study but generated an AAV.CMV.null.WPRE.io2 vector (Figure S1) using restriction digest to remove the confounding foreign transgene (GFP) sequence from AAV.CMV.GFP.WPRE.io2. AAV vectors were packaged into AAV2 capsids and purified (CsCl density gradient) by VectorBuilder (Edinburgh, UK). The final product was concentrated in PBS supplemented with 200 mM NaCl and 0.001% pluronic F-68. Viral titer (1.63E13 gc/mL) was determined by VectorBuilder using qPCR of the inverted terminal repeat. The virus is provided with a certificate of analysis confirming negative sterility and mycoplasma tests and purity confirmed as >80%. Endotoxin levels are guaranteed <10 endotoxin units per milliliter.

### Histology and microscopy

For tissue sectioning, single eyes were enucleated and fixed for 24 h in 1% cytofix/cytoperm (554714, BD Biosciences, Oxford, UK) at 4°C, before transferring them to 20% sucrose (84100-250G, Sigma-Aldrich) solution in Dulbecco’s PBS (D8537, Sigma-Aldrich) for a further 24 h at 4°C. Eyes were then frozen on dry ice in optimal cutting temperature solution (LAMB/OCT, Thermo Fisher Scientific, Oxford, UK) and stored at −70°C until sectioning. Using a Leica CM3050S cryostat, 12-μm-thick sections were made onto Epredia Superfrost Plus slides (MIC3040; Scientific Laboratory Supplies, Nottingham, UK) and stored at −20°C.

For flat mount staining, eyes were fixed for 30 min on ice in 4% paraformaldehyde (16% diluted in PBS; 28908, Thermo Fisher Scientific) before a further fixation in 1% cytofix/cytoperm for 24 h at 4°C. Eyes were then dissected into posterior cups and retinas separated from RPE/choroid before blocking in 1× BD perm/wash (554714, BD Biosciences) with purified rat anti-mouse CD16/32 Fc block (clone 2.4G2; 553142, BD Biosciences) 1 in 100 and 5% normal donkey serum for 24 h at room temperature. Primary antibodies (Table S26) were diluted in 1× BD perm/wash and incubated at room temperature for 72 h. Tissue was washed 3× in PBS for 1 h before incubating with secondary antibody diluted in 1× BD perm/wash (Table S26) for 24 h at room temperature. Tissue was mounted in Ce3d solution (427704, BioLegend, London, UK) and imaged on a Leica SP5II.

For section staining, tissue was blocked (purified rat anti-mouse CD16/32 Fc block diluted 1 in 100 with 1% bovine serum albumin in PBS) for 1 h at room temperature and primary antibody (Table S26) was added at 4°C overnight. Tissue was washed 3× in PBS before incubation with relevant secondary antibodies (Table S26) for 2 h at room temperature. Following a further three washes in PBS, DAPI (Table S26) was added (1 in 10,000 in PBS) for 5 min at room temperature before mounting in VectaShield anti-fade medium (H1000, Vector Laboratories, Newark, CA). Images were captured using a Leica SP8.

For ONL and INL thickness analysis, sections were stained for 5 min at room temperature in DAPI (1 in 10,000 in PBS) before mounting in anti-fade mounting medium. Images were captured at 10× magnification on an EVOS FL (Invitrogen, Oxford, UK).

ONL and INL thickness analysis and image processing were performed using Fiji.

### Flow cytometry

Following enucleation, single eyes were dissected using a limbal incision to remove the posterior segment. Whole retina and vitreous were extracted and together with 100 μL ice-cold PBS transferred into a 1.5-mL microfuge tube. The tissue was mechanically dissociated by rapping the tube across an 80-well standard rack. Samples were then passed through a 96-well 60-μm nylon mesh filter plate (Merck Millipore, Watford, UK) by centrifugation at 400 × *g* for 5 min. Supernatant was discarded, and the cell pellet was resuspended in cell staining buffer (420201, BioLegend) before transfer to a 96-well V-bottom plate for staining. Spleen and brain were mechanically dissociated with a syringe plunger and passed through a 70-μm cell strainer with ice-cold PBS. Cells were pelleted at 400 × *g* for 3 min and resuspended in ammonium-chloride-potassium buffer to lyse erythrocytes before pelleting and a final re-suspension in FACS buffer.

To stain samples, cells were first blocked with purified rat anti-mouse CD16/32 Fc block (clone 2.4G2; 553142, BD Biosciences) diluted (1 in 50) in FACS buffer for 20 min at 4°C, followed immediately by addition and incubation with antibodies (Table S26) for 30 min at 4°C. For the identification of live populations, cells were additionally stained with DAPI (included with antibodies in the total stain) or 7AAD (added in FACS buffer after antibody stain; Table S26).

A four-laser Fortessa X-20 flow cytometer (BD Cytometry Systems, Oxford, UK) was used to acquire cell suspensions, and compensation was performed using OneComp eBeads (Thermo Fisher Scientific). For compensation of tdTomato, cell suspensions were prepared from *Cx3cr1CreER26-tdTomato*^*+/−*^ brains. Fluorescence-minus-one controls were used to assist in the gating of microglia markers. For the analysis of inflammation in aging animals (Figure 5), an LSR II flow cytometer (BD Cytometry Systems) was used to acquire samples. A standard curve was generated with seven 2-fold serial dilutions of a known concentration of splenocytes (top standard 20E6), which were acquired on the flow cytometer in a similar manner to the samples, allowing for calculation of absolute counts.^63^ Data were analyzed using FlowJo software (Tree Star, San Carlos, CA). Single retinas were used for flow cytometric analysis, and contralateral eyes were fixed for flat mount or section tissue staining.

### Fluorescent-activated cell sorting

Single eyes were processed from naive (*n* = 3–4 of each sex and age or *n* = 2 for 9-month-old males), day 12 (*n* = 3–4), and day 28 (*n* = 3–4) post-AAV injection. Single-cell suspensions from retinas were made, as described earlier and re-suspended in 250 μL FACS buffer. Microglial cells (tdTom^hi^ and DRAQ7) were sorted immediately after the addition of DRAQ7 (one drop; DR77524; Biostatus, Shepshed, UK) using a BD Influx Cell Sorter. Samples were prepared in small batches to maintain cell viability and ensure high-quality RNA.

From each sample, 600 cells were sorted directly into 0.2-mL endonuclease-free tubes containing 0.05 μL RNase inhibitor, 0.95 μL lysis buffer, and 7.5 μL nuclease-free water (previously optimized^21^), using reagents from SMART-Seq version 4 Ultra Low Input RNA Kit for Sequencing (Takara Bio USA, Mountain View, CA). Each batch was randomized to include a mix of all conditions to mitigate the batch effects of sort and cDNA preparation. Samples were collected over 2 days, with all samples collected in 1 day processed to cDNA preparations together (sorted samples were stored in lysis buffer on ice until all samples were collected). Contralateral eyes were either used for flow cytometry analysis (LSR II, Figure 5) or fixed for tissue staining (Figures 1 and 6).

### RNA-seq

The SMART-Seq version 4 Ultra Low Input RNA Kit for Sequencing was used for cDNA preparation and amplification (16 PCR cycles), according to the user manual, before cDNA isolation using the Agencourt AMPure XP Kit (Beckman Coulter, Brea, CA). Quantification of cDNA was performed using the Agilent High Sensitivity DNA Kit on an Agilent 2100 Bioanalyser (Agilent Technologies, Santa Clara, CA), and 150 pg of cDNA was used for library preparation using the Nextera XT DNA Library Preparation Kit (Illumina, San Diego, CA).

The Illumina NextSeq 500/550 High Output version 2 kit (75 cycles) on an Illumina NextSeq 500 Sequencing System was used to sequence samples to depths of up to 2.9 million single-end 75-nt length reads per sample. NextSeq Control Software version 2.0 and Real-Time Analysis Software version 2 were used for image analysis, base calling, and generation of sequence reads before conversion to FASTQ files using the bcl2fastq2 version 2.20 software (Illumina), producing four FASTQ files per sample.

### Bioinformatics

Data were processed to read counts using Galaxy^64^ before exporting for further differential expression analysis performed in R studio (version 2022.12.0 + 353 and R version 4.2.2; Figure S2). Sequencing data were first assessed by FASTQC^65^ and data were compiled using multiQC (version 1.11; Figure S2) before mapping using STAR (version 2.7.8a) and reference index mm10. All samples produced high-quality sequencing data, with the majority of samples achieving >80% in unique mapping, and the lowest achieved was 73.6%. Resulting bam files were concatenated using mergeSAMfiles (version 2.18.2.1), and read counts were determined using feature counts (version 2.0.1) with refGene (UCSC genome browser database: https://hgdownload.soe.ucsc.edu/goldenPath/mm10/bigZips/genes/) for annotation. ComBat_seq in sva (Surrogate Variable Analysis, version 3.46.0) was used to remove batch variation from sample collection (Figure S2) before applying DESeq2 (version 1.38.0) for differential expression analysis (log fold change [LFC] and *p* adjusted values applied are reported in the supplemental tables). For gene enrichment analysis, webgestalt^66^ was used to assess over-representation analysis in GO by BP terms. The full protein-coding genome was used as a reference gene list, and standard parameters were used, including the Benjamini-Hochberg test for multiple testing. PCA plots and heatmaps were produced in R Studio. Heatmaps were generated from the *Z* scores of transformed data (variance stabilizing transformation, DESeq2).

### qPCR

Remaining cDNA preparation from the SMARTSeq kit was used as input to validate the sequencing platform (Figure S3). Samples were analyzed using PowerUp SYBR Green Master Mix (A25742; Thermo Fisher Scientific), with 1 ng cDNA and 500 nM of each primer as input (list of primers in Table S27). All samples were run in duplicate on a Quantstudio 3 Real-Time PCR system (A28137; Thermo Fisher Scientific).

### Statistical analysis

All remaining statistical analyses were performed in GraphPad Prism (version 9.4.0, GraphPad Software, San Diego, CA). For analysis of flow cytometric data, a two-way ANOVA with independent factors treatment and sex or treatment and age was applied. Where significant effects were identified by two-way ANOVA, the Holm-Šídák test was used for further multiple comparison. Where significance was seen for treatment only, then a direct comparison of treatments was assessed (separated by sex), if row and column were both *p* < 0.1 by two-way ANOVA, all terms were compared. Each time point was compared to naive controls in separate tests. No significant differences were observed comparing baseline retinal thickness between ages, and thus when treatment groups were added, statistical analysis was performed for each age separately, to keep two independent factors (sex and treatment). For the aging flow cytometric data, significant differences were observed for treatment compared to age-matched naive eyes; however, for simplicity and to focus on age effect, only the significant differences between ages are displayed in the figure. Simple linear regression was used to compare CD3 count to ONL thickness. All data in graphs and text are expressed as mean ±SD. A Fisher’s exact test was used to compare frequency distribution of clinical presentation variables (male vs. female), such as PVS, cellular infiltrate, and retinal folds.

## Data and code availability

The RNA-seq dataset generated in this study can be found at the Gene Expression Omnibus repository, accession number GSE266332. All other raw data will be made available by authors at request.

## Acknowledgments

The research was supported by the 10.13039/501100000272National Institute for Health and Care Research (NIHR) Biomedical Research Center based at Moorfields Eye Hospital NHS Foundation Trust and UCL Institute of Ophthalmology (BRC4-03-RB414-405), the 10.13039/100010269Wellcome Trust (Institutional Translation Partnership Award Fellowship; 222064/Z/20/Z), the 10.13039/501100000265Medical Research Council (Impact Acceleration Account; MR_X502856_1), Sight Research UK (SAC054) and The Underwood Trust. The authors wish to thank Andrew Herman, Poppy Miller, Celyn Dugdale, Helen Rice (Flow Cytometry Facility, University of Bristol), Christy Waterfall and Jane Coghill (Genomics Facility, University of Bristol), the Wolfson Bioimaging Facility, and the University of Bristol Animal Services Unit for their technical assistance.

## Author contributions

A.J.C., P.M.L., A.W., and D.A.C. performed the experiments. Y.K.C. provided materials. A.J.C., P.M.L., Y.K.C., A.D.D., and D.A.C. planned the experiments. A.J.C. analyzed the data. A.J.C., A.D.D., and D.A.C. drafted the manuscript. A.J.C., Y.K.C., A.D.D., and D.A.C. revised and edited the manuscript.

## Declaration of interests

The authors declare no competing interests.

## Supplemental information

Document S2. Tables S1–S25

Document S3. Article plus supplemental information
