# Supplementary material for: Characterization of the ocular inflammatory response to AAV reveals divergence by sex and age
Source: Mol Ther. 2025 Jan 17;33(3):1246–63. doi: 10.1016/j.ymthe.2025.01.028 (PMC11897812; doi:10.1016/j.ymthe.2025.01.028)
Supplement: Document S1. Figures S1–S11 and Tables S26 and S27 [file mmc1.pdf]

YMTHE, Volume *33*

Supplemental Information

Characterization of the ocular inflammatory response to AAV reveals divergence by sex and age

Alison J. Clare, Philip M. Langer, Amy Ward, Ying Kai Chan, Andrew D. Dick, and David A. Copland

Supplemental Figures

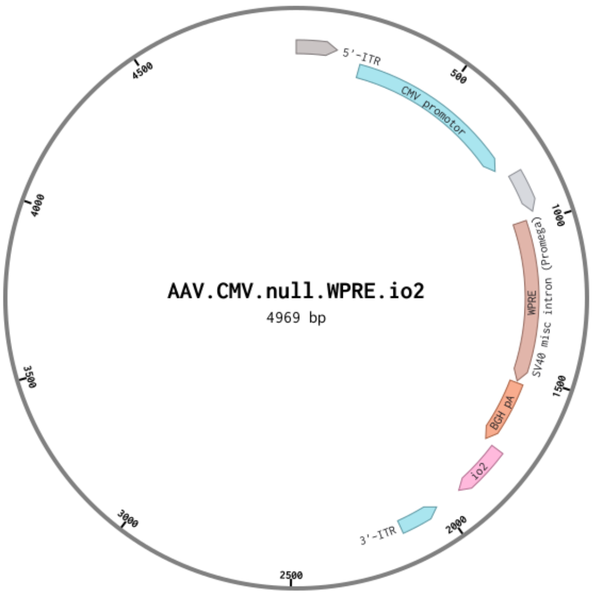

Figure S1. AAV plasmid map. Plasmid map for AAV2.CMV.null.WPRE.io2 vector. CMV; cytomegalovirus, WPRE; woodchuck hepatitis virus post-transcriptional response element, bGH pA; bovine growth hormone polyadenylation signal.

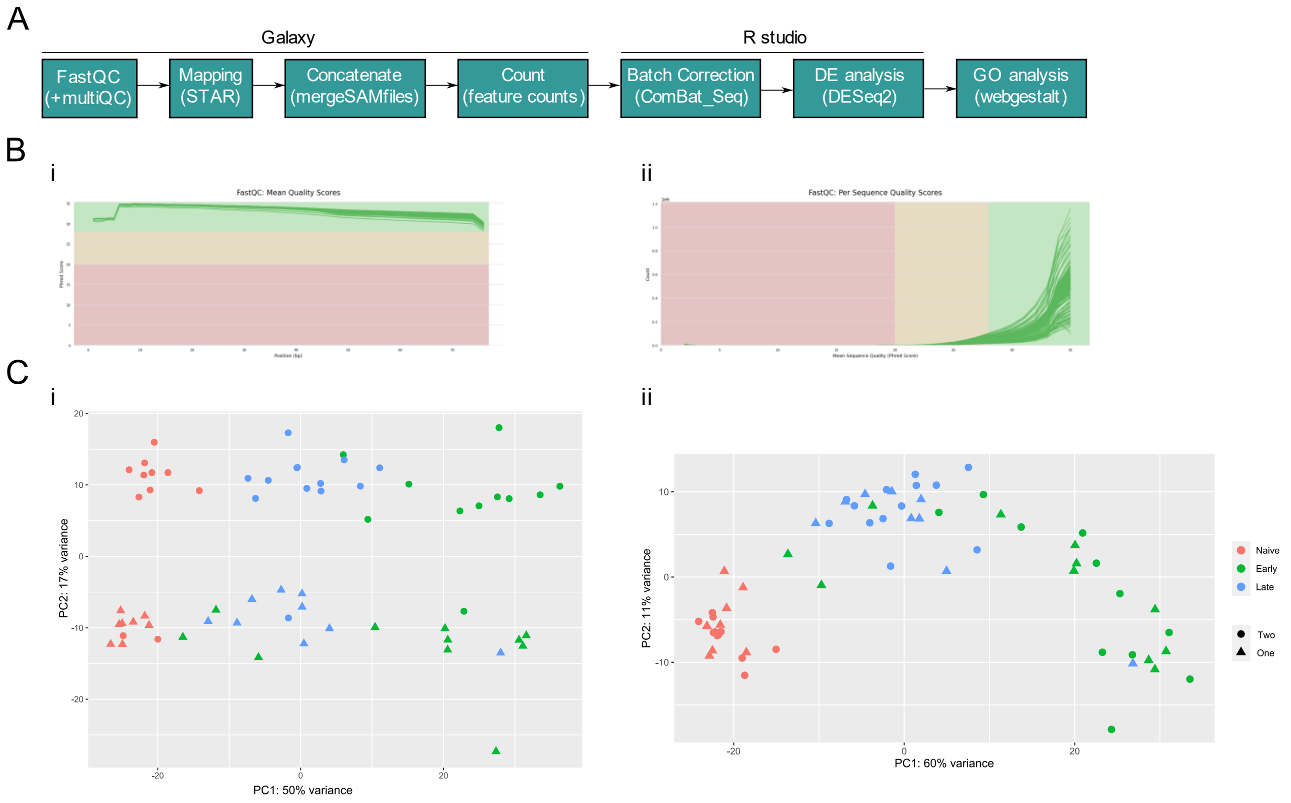

Figure S2. RNA-seq data processing. (A) The bioinformatics pipeline applied to process RNA-seq data. (B) FastQC/multiQC analysis revealed Phred quality scores >30 in all base positions (i), and a mean Phred score >30 for most reads (ii). (C) PCA clustering analysis identifies a separation by batch, indicating variation by batch collection (group 1 vs group 2) (i). This is corrected using combatSeq (ii).

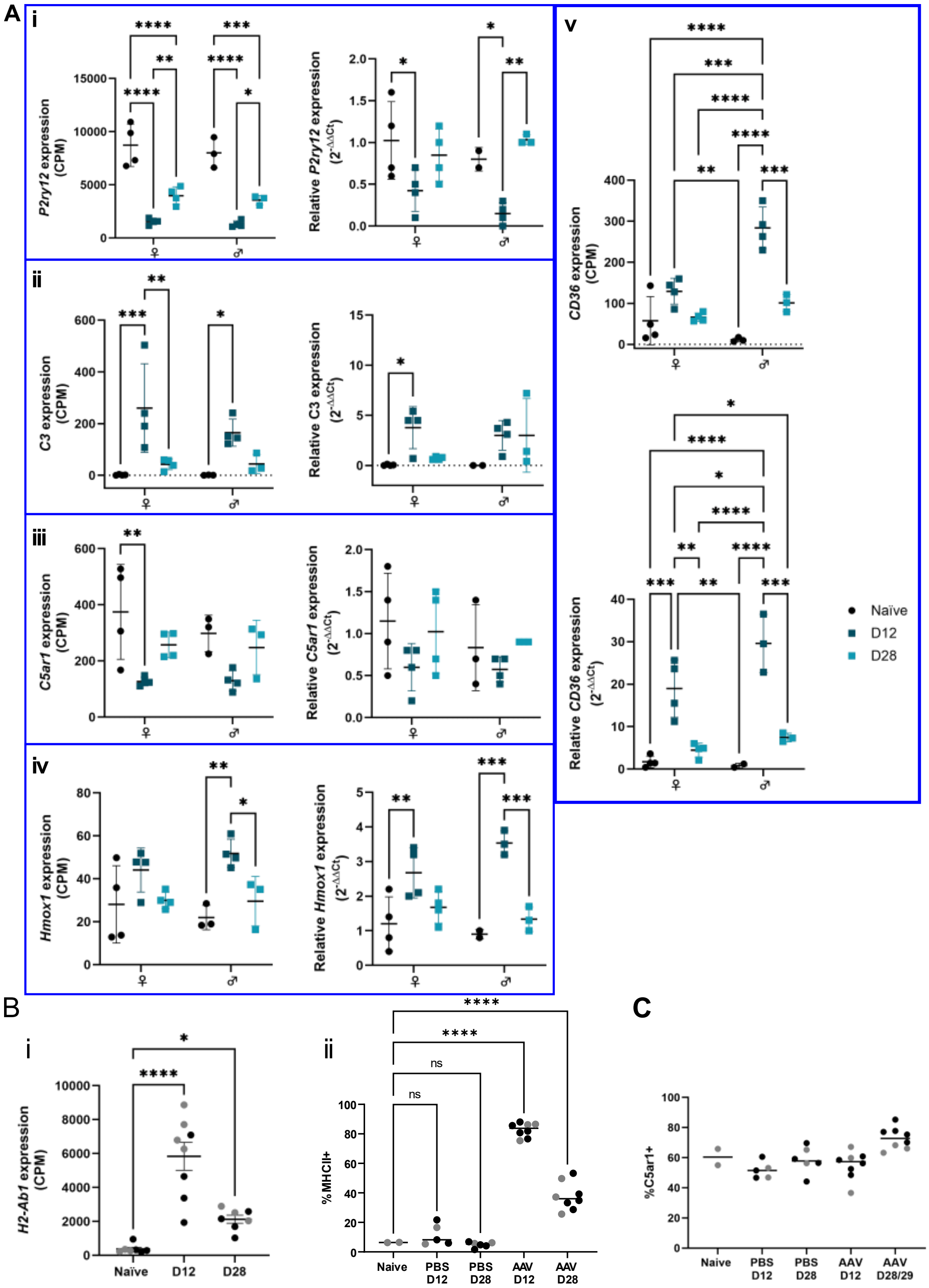

Figure S3. Quantitative PCR validation of RNA-seqeuncing data. (A) RNA-seq counts per million and qPCR relative quantification for *P2ry12* (i), *C3* (ii), *C5ar1* (iii), *Hmox1* (iv), *CD36* (v). (B) Comparison of RNA-seq counts per million of gene product H2-Ab1 (i) and flow cytometric analysis of protein equivalent IA/IE (MHCII) expression on microglia (ii). (C) Flow cytometric analysis of C5ar1 protein expression on microglia.

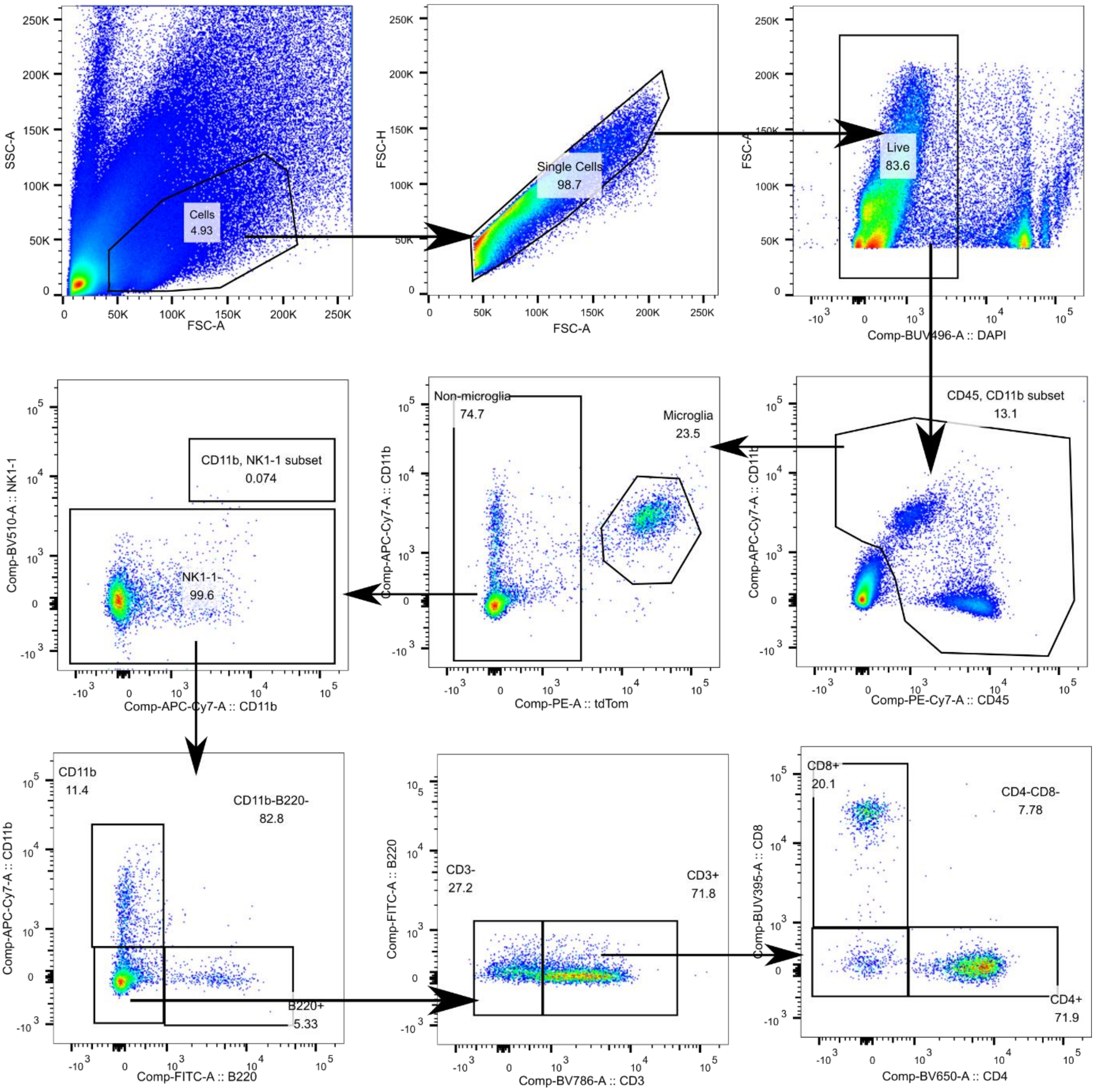

Figure S4. Gating strategy for flow cytometric analyses. Retinas were gated for cells, singlets, live cells, and immune cells. From here, they were separated into microglia (tdTomato+) and non-microglial cells (tdTomato-). Microglia cells were then assessed for % expression of different markers (e.g. CD44, BST2, P2RY12). Non-microglia cells were separated into myeloid cells (CD11b+), B cells (B220+ FITC or CD19+ BV421), and other (B220-, CD11b-). T cells were identified from B220-Cd11b- using CD3 and then further discriminated into CD4+, CD8+ and CD4-CD8-.

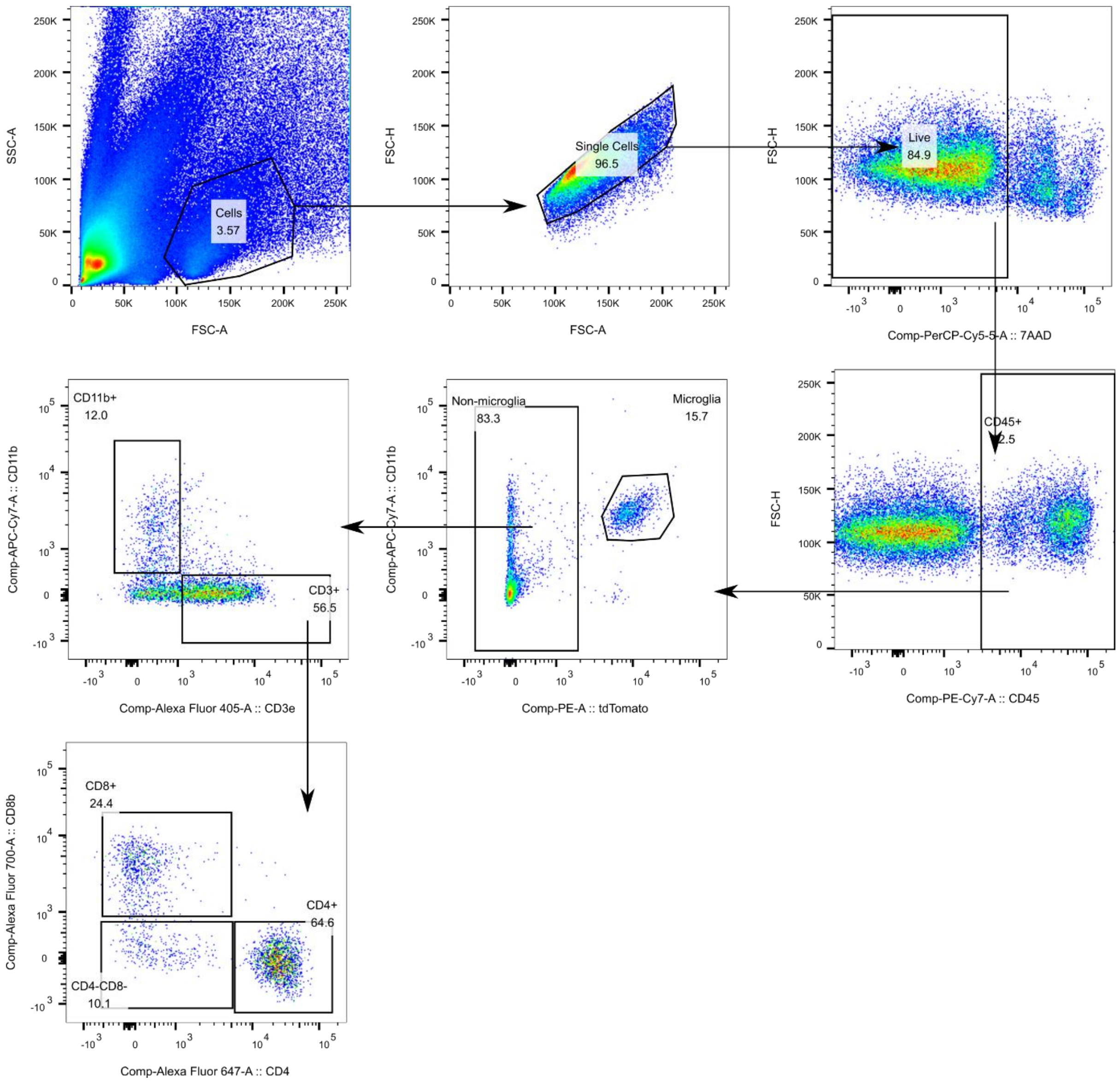

Figure S5. Gating strategy for flow cytometry in Figure 5. Retinas were gated for cells, singlets, live cells, and CD45+ cells. From here, they were separated into microglia (tdTomato+CD11b+) and non-microglial cells (tdTomato-). Non-microglia cells were separated into myeloid cells (CD11b+), and T cells (CD3). T cells were further categorised into CD4+, CD8+ and CD4-CD8-.

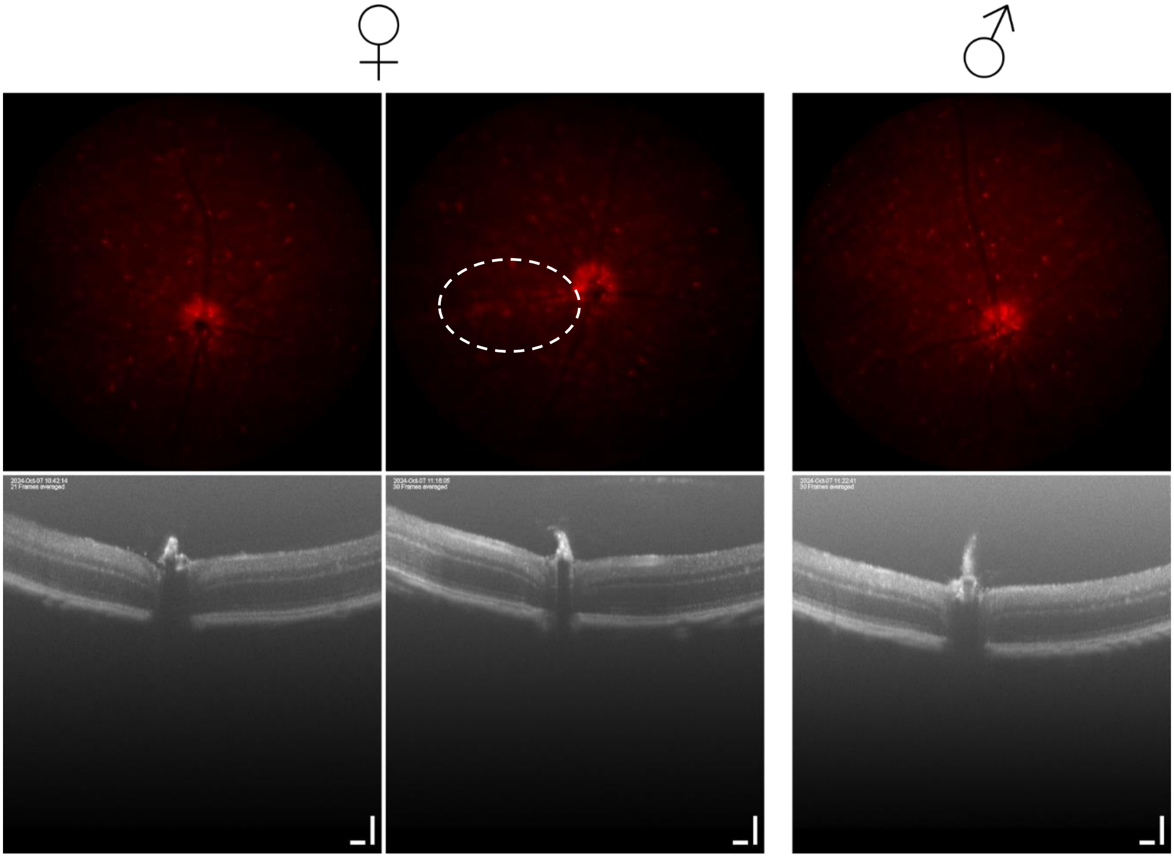

Figure S6. PBS injected eyes show no signs of inflammation. Female and Male representative eyes from PBS injected control animals at the equivalent timepoint for peak inflammation for AAV injected eyes(D10-12). No significant perivascular sheathing (PVS), except for one female with mild PVS (white dashed circle) and no infiltrating cells observed on OCT scans. Scale bar is 100 µm.

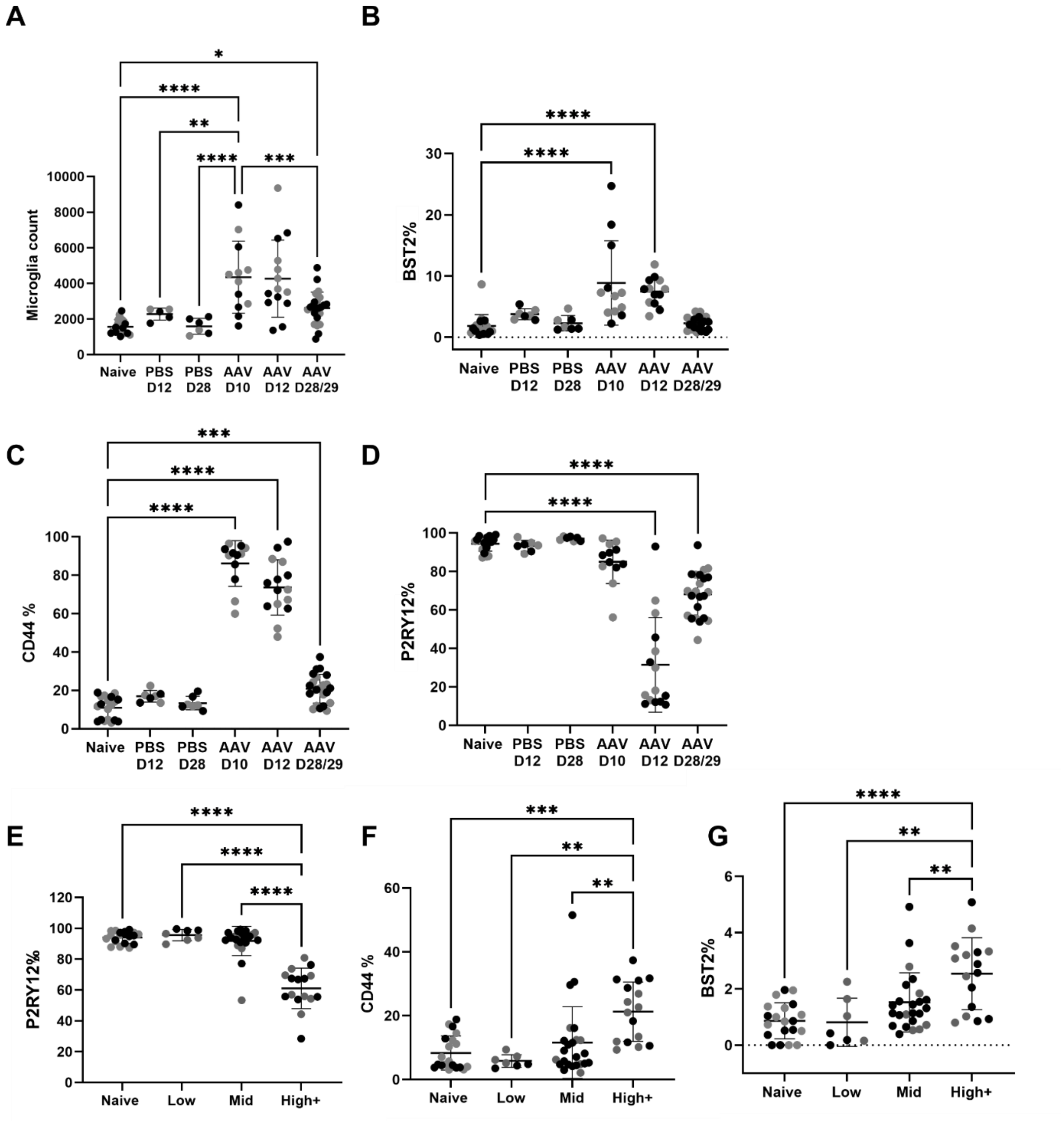

Figure S7. Microglial changes are AAV specific. Flow cytometric analyses of Naïve eyes and AAV and PBS injected eyes at D12 and D28/29 post injecton. (A) Microglia count. Microglial expression of BST2 (B), CD44 (C) and P2RY12 (D). Comparison of microglial activation marker expression at D29 post AAV injectionwith increasing AAV dose for (E) P2RY12, (F) CD44 and (G) BST2. Low dose (2e7 gc/eye), mid dose (2e8 gc/eye) and high+ dose (1e10 gc/eye). Females = black dots and males = grey dots.

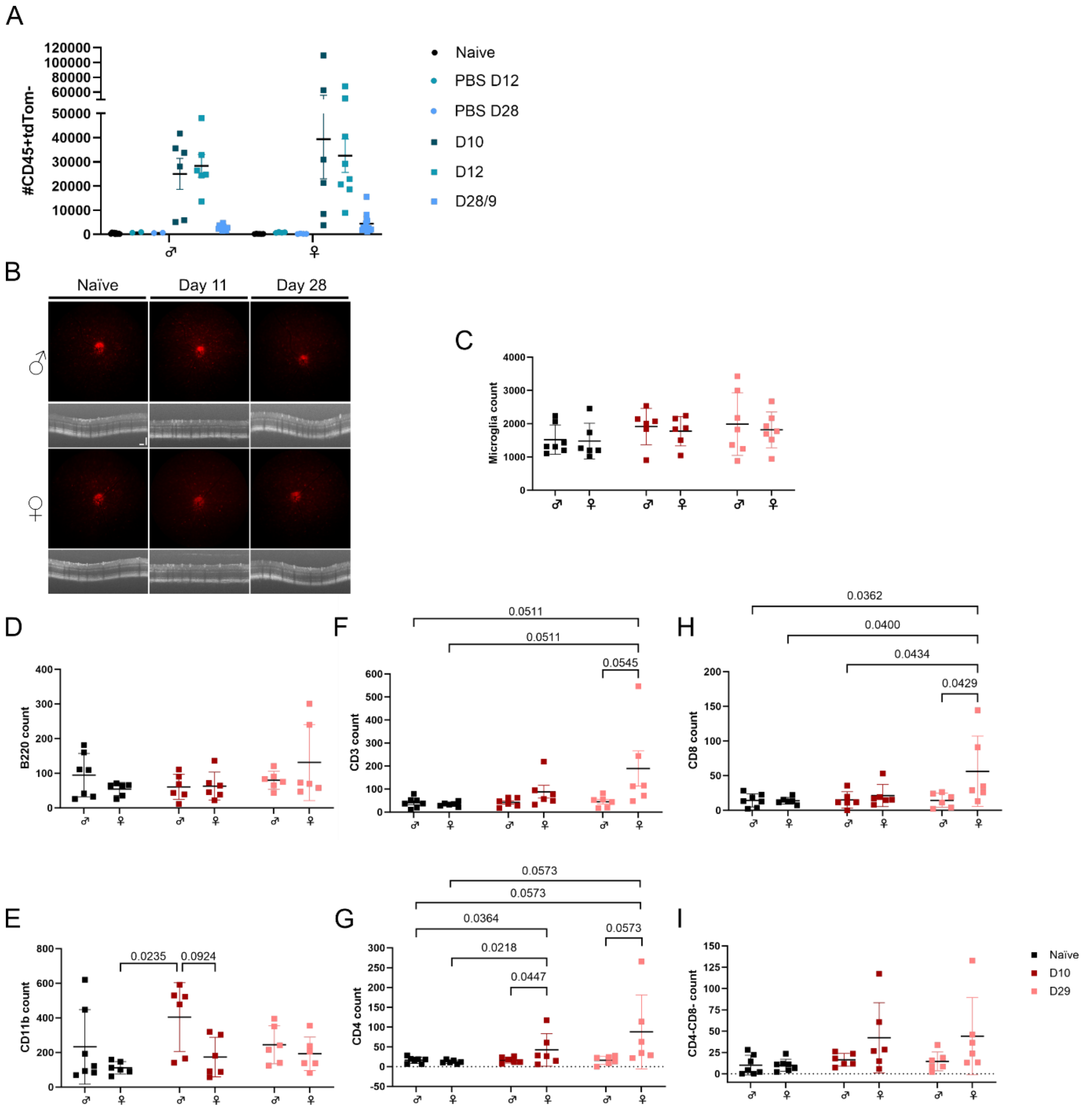

Figure S8. Ocular immune response to AAV. (A) CD45+tdTom- cell count with AAV2 and PBS injected eyes. (B) Clinical imaging (tdTomato fundus fluorescence and OCT) after injection of lower (2E8 gc/eye) AAV2 dose. Scale bar is 100 µm. Flow cytometric analysis of immune cells in retina after low dose (2E8 gc/eye) of AAV2. Graphs show microglia count (C) B cells (B220+; D), CD11b+ cells (E), CD3+ cells (T cells) (F), CD3+CD4+, (G) CD3+CD8+, (H) and CD3+CD4-CD8- cells (I). Statistic analysis performed by two-way ANOVA with Holm-Sidak multiple correction.

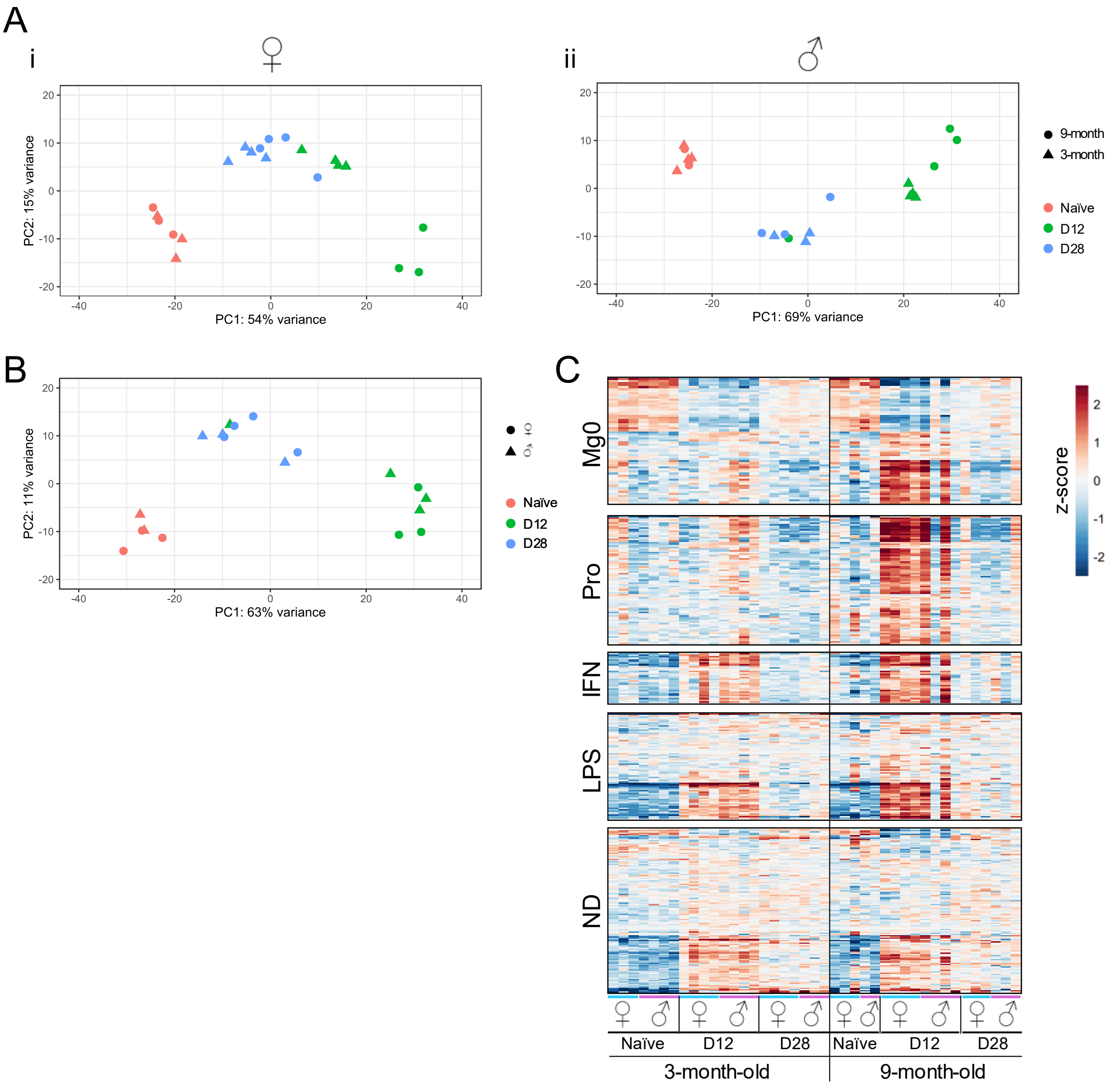

Figure S9. RNA-sequencing analysis of the microglia response to AAV in 9-month versus 3-month-old mice. PCA clustering analysis of naïve, D12 and D28 post AAV delivery (intravitreal; IVT) for comparison of 3 and 9-month-old females (Ai), males (Aii) and comparison of 9-month-old males and females (B). Heatmap of curated activated microglia gene modules (Friedman et al. 2018). Mg0, microglia resting state; Pro, proliferation; IFN, interferon response; LPS, response to lipopolysaccharide; ND, neurodegeneration.

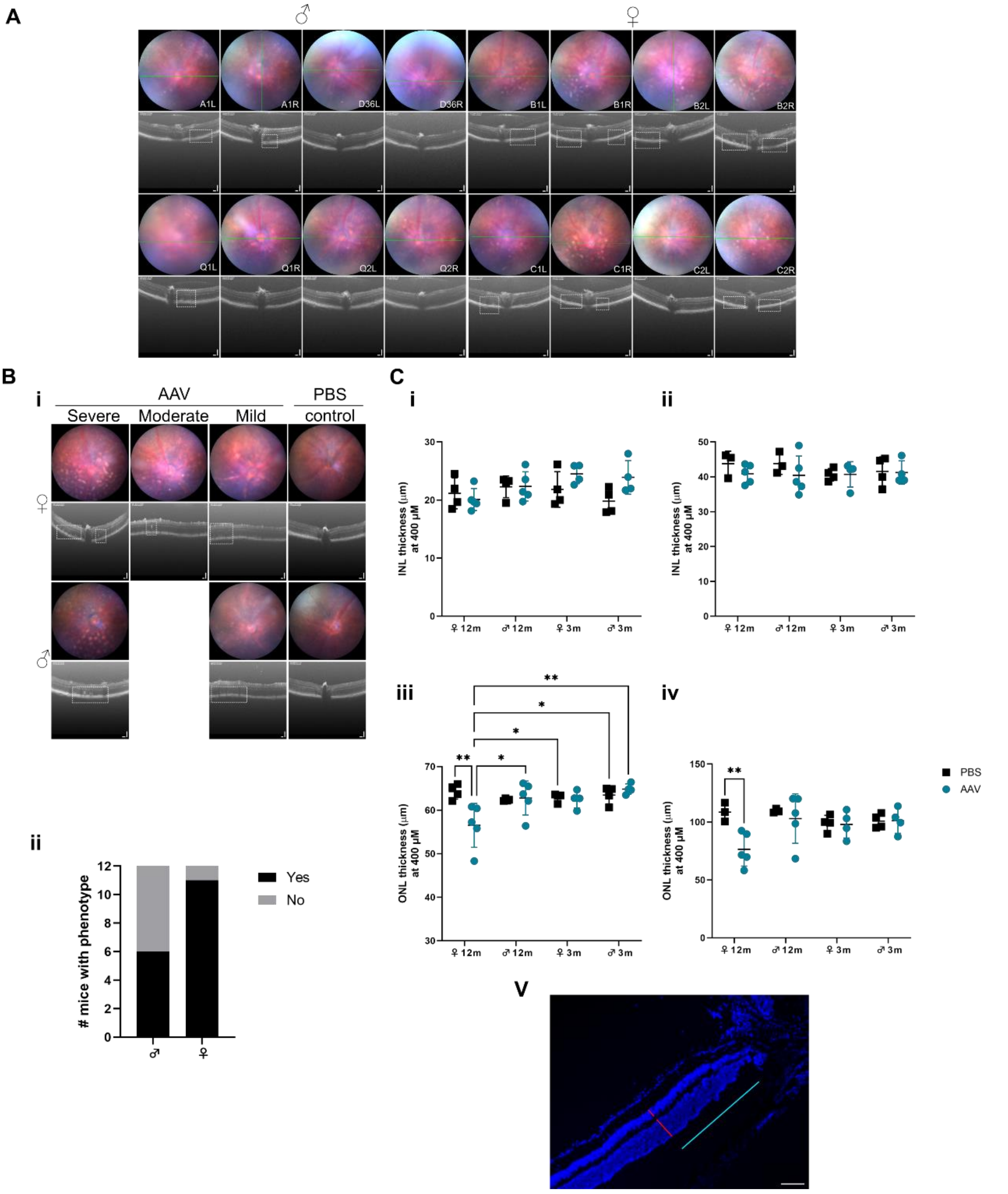

Figure S10. Degenerative phenotypes in aged animals. (A) All fundus and OCT images from 18-month-old tdTom mice. Signs of retinal folds indicated by white dashed line box. (B) Clinical phenotypes observed in C57BL6/J 11 and 12-month-old mice, ranging in severity (i) and yes/no scoring of presence of phenotype (ii). (C) Graph shows INL thickness, as measured from OCT images (i) and ex vivo histology (ii) and ONL thickness from OCT images (iii) and by ex vivo histology (iv). For ex vivo analysis, sections were stained with DAPI and 400 µm distance measured from optic nerve (blue line) before taking measurements of ONL and INL (red lines) (v). Scale bar is 100 µm.

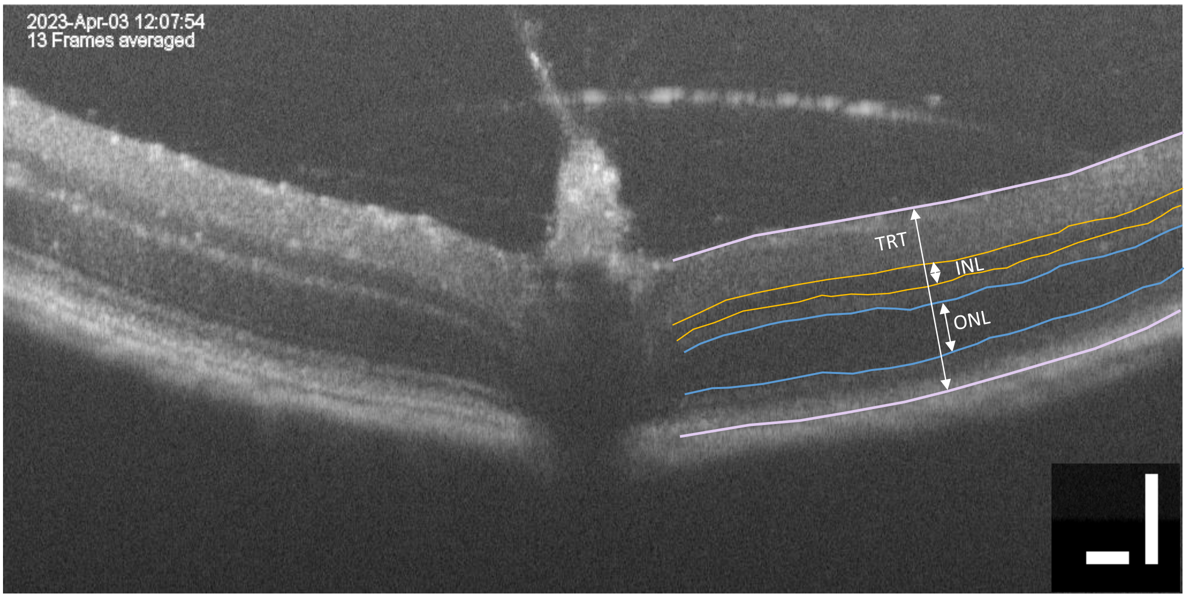

Figure S11. Example of segmentation in optical coherence tomography image for thickness analysis. Purple lines and arrow indicate top and bottom points for total retinal thickness (TRT) measurement. Yellow lines and arrow indicate measurement points for inner nuclear layer thickness. Blue line and arrow indicate points for measurement of outer nuclear layer thickness. Scale bar is 100 µm.

Table S1. Male differentially expressed genes at D12 compared to naïve eyes (DESeq2 analysis; LFC>1, padj<0.05)

Table S2. Male differentially expressed genes at D28 compared to naïve eyes (DESeq2 analysis; LFC>1, padj<0.052

Table S3. Female differentially expressed genes at D12 compared to naïve eyes (DESeq2 analysis; LFC>1, padj<0.05)

Table S4. Female differentially expressed genes at D28 compared to naïve eyes (DESeq2 analysis; LFC>1, padj<0.05)

Table S5. Significantly enriched gene ontology terms (biological process) in the male only DEGs changed at D12

Table S6. Female differentially expressed genes in 9-month isolated microglia compared to 3-month-old at D12 (DESeq2 analysis; LFC>0, padj<0.1)

Table S7. Significantly enriched gene ontology terms (biological process) in the upregulated genes at D12 in 9-month female microglia compared to 3-month-old

Table S8. Male differentially expressed genes in 9-month isolated microglia compared to 3-month-old at D12 (DESeq2 analysis; LFC>0, padj<0.1)

Table S9. Significantly enriched gene ontology terms (biological process) in the upregulated genes at D12 in 9-month male microglia compared to 3-month-old

Table S10. Male differentially expressed genes in 9-month isolated microglia compared to 3-month-old at D28 (DESeq2 analysis; LFC>0, padj<0.1)

Table S11. Female differentially expressed genes in 9-month isolated microglia compared to 3-month-old at D28 (DESeq2 analysis; LFC>0, padj<0.1)

Table S12. Genes differentially expressed at D12 by age in males (3-month vs 18-month; DESeq2 analysis; LFC>0, padj<0.1)

Table S13. Genes differentially expressed at D28 by age in males (3-month vs 18-month; DESeq2 analysis; LFC>0, padj<0.1)

Table S14. Genes differentially expressed at D12 by age in females (3-month vs 18-month; DESeq2 analysis; LFC>0, padj<0.1)

Table S15. Genes differentially expressed at D28 by age in females (3-month vs 18-month; DESeq2 analysis; LFC>0, padj<0.1)

Table S16. Significantly enriched gene ontology terms (biological process) in the genes differentially expressed at D12 by age in females (3-month vs 18-month)

Table S17. Female differentially expressed genes in 18-month isolated microglia compared to 3-month-old at D12 (DESeq2 analysis; LFC>0, padj<0.1)

Table S18. Male differentially expressed genes in 18-month isolated microglia compared to 3-month-old at D12 (DESeq2 analysis; LFC>0, padj<0.1)

Table S19. Significantly enriched gene ontology terms (biological process) in the upregulated genes at D12 in 18-month male microglia compared to 3-month-old

Table S20. Significantly enriched gene ontology terms (biological process) in the upregulated genes at D28 in 18-month male microglia compared to 3-month-old

Table S21. Significantly enriched gene ontology terms (biological process) in the upregulated genes at D12 in 18-month female microglia compared to 3-month-old

Table S22. Significantly enriched gene ontology terms (biological process) in the upregulated genes at D12 in 18-month female microglia compared to 3-month-old

Table S23. Female differentially expressed genes in 18-month isolated microglia compared to 3-month-old at D28 (DESeq2 analysis; LFC>0, padj<0.1)

Table S24. Significantly enriched gene ontology terms (biological process) in the upregulated genes at D28 in 18-month female microglia compared to 3-month-old

Table S25. Male differentially expressed genes in 18-month isolated microglia compared to 3-month-old at D28 (DESeq2 analysis; LFC>0, padj<0.1)

Table S26. Antibodies and live/dead stain used in flow cytometry and immunohistochemistry

*used as B cell marker at time-points D12 and 28

**used as validation markers at time-points D12 and D28

***IB4 in flatmounts only, DAPI used in sections only

Use	Target	Fluorophore	Clone	Catalog #	Company	Dilution
Fortessa panel Fig 2 and 4	CD45	PE-cy7	30-F11	552848	BD Biosciences	1 in 1500
	CD11b	APC-cy7	M1/70	557657	BD Biosciences	1 in 200
	CD3	BV785	145-2c11	100355	Biolegend	1 in 40
	CD4	BV650	RM4-5	100546	Biolegend	1 in 200
	CD8	BUV395	H35-17.2	740278	BD Biosciences	1 in 400
	B220	FITC	RA3-6B2	553088	BD Biosciences	1 in 200
	CD19*	BV421			Biolegend	1 in 200
	NK1.1	BV510	PK136	108738	Biolegend	1 in 500
	CD44	SB600	RM7	63-0441-82	Thermo Fisher Scientific	1 in 200
	Bst2	PE-efluor610	eBio927	61-3172-82	eBiosciences	1 in 200
	P2ry12	APC	S16007D	848006	Biolegend	1 in 200
	I-A/I-E (MHCII)**	BV711	M5/114.15.2	107643	Biolegend	1 in 200
	C5ar1**	PerCP-eFluor710	20/70	135813	Biolegend	1 in 400
	Live/Dead stain: DAPI	BUV496	n/a	564907	BD Biosciences	1 in 10000
LSR II panel Fig 5	CD45	PE-cy7	30-F11	552848	BD Biosciences	1 in 1500
	CD11b	APC-cy7	M1/70	557657	BD Biosciences	1 in 200
	CD3e	BV421	145-2c11	100341	Biolegend	1 in 40
	CD4	APC	RM4-5	100516	Biolegend	1 in 200
	CD8	AF700	YTS156.7.7	126617	Biolegend	1 in 200
	Live/Dead stain: 7AAD	PE-Cy5	n/a	A1310	Thermo Fisher Scientific	1 in 400
IHC Fig 1 and 7	CD3	AF488	17A2	100210	Biolegend	1 in 200
	RFP	n/a	n/a	600-401-379	Rockland	1 in 300
	Isolectin-B4-HRP***	n/a	n/a	L2140	Sigma-Aldrich	1 in 500
	Goat anti-rabbit secondary (with RFP)	AF555	n/a	A-21429	Invitrogen	1 in 1000
	(with IB4)	efluor450	n/a	48-4317-82	Invitrogen	1 in 500
	DAPI***	n/a	n/a	MBD0015	Sigma	1 in 10000

Table S27. Primers used in quantitative PCR validation

Primer target	Forward	Reverse
β-actin	AGCCATGTACGTAGCCATCC	CTCTCAGCTGTGGTGGTGAA
P2ry12	GGGTCACAGTGCAAGAACAC	AAGGTGGTATTGGCTGAGGTG
C3	GCTTCAGGGTCCCAGCTACTA	TGTCCACTGGCTCCTGTCAA
C5ar1	ATGGACCCCATAGATAACAGCA	GAGTAGATGATAAGGGCTGCAAC
Hmox1	ACCCAGTCTATGCCCCACTC	CCAGTGAGGCCCATACCAGA
CD36	ATGGGCTGTGATCGGAACTG	GTCTTCCCAATAAGCATGTCTCC
